# Reducing Wallacean shortfalls for the coralsnakes of the *Micrurus lemniscatus* species complex: Present and future distributions under a changing climate

**DOI:** 10.1371/journal.pone.0205164

**Published:** 2018-11-14

**Authors:** Levi Carina Terribile, Darlan Tavares Feitosa, Matheus Godoy Pires, Paula Carolina Rodrigues de Almeida, Guilherme de Oliveira, José Alexandre Felizola Diniz-Filho, Nelson Jorge da Silva

**Affiliations:** 1 Instituto de Biociências, Universidade Federal de Goiás, UFG, Regional Jataí, Brazil; 2 Programa de Pós-Graduação em Ciências Ambientais e Saúde, Escola de Ciências Médicas, Farmacêuticas e Biomédicas, Pontifícia Universidade Católica de Goiás, Goiânia, Goiás, Brazil; 3 Programa de Pós-Graduação em Biotecnologia e Biodiversidade–Rede Ampla, Universidade Federal de Goiás, Brazil; 4 Centro de Ciências Agrárias, Ambientais e Biológicas, Universidade Federal do Recôncavo da Bahia, Bahia, Brazil; 5 Departamento de Ecologia, Universidade Federal de Goiás, UFG, Brazil; Universita degli Studi di Napoli Federico II, ITALY

## Abstract

South American coralsnakes are characterized by inconspicuous and poorly known species, which are potentially very sensitive to climate change. Here, we assess the impact of future climate change on the distributions of the *Micrurus lemniscatus* species complex after addressing the Wallacean shortfalls and refining the knowledge about their current geographic distributions. We also evaluate the efficiency of the current reserve network to protect the species in the present and future. We applied ecological niche model tools through a carefully examined set of occurrence records to generate potential present distributions and to project these distributions into future scenarios of climate change. Specific thresholds based on occurrence records along with expert opinions were used to delineate the geographic distribution of each species. A hierarchical ANOVA was applied to evaluate the uncertainties in species distributions across niche modeling methods and climate models and nested into the time factor (present and future). Multiple regression models were used to infer the relative importance of the climatic variables to determine the species’ suitability. A gap analysis was performed to address the representativeness of species distributions into protected areas. Predicted geographic distributions were compatible with the known distributions and the expert opinions, except for *M*. *l*. *carvalhoi*. New areas for field research were identified. Variation in precipitation was the most important factor defining the habitat suitability for all species, except for *M*. *diutius*. All taxa (except *M*. *l*. *lemniscatus*) will shrink their distributions in the future; less than 50% of the present suitable areas are protected in reserve networks, and less than 40% of these areas will be held in reserves in the future. We found strong evidence that coralsnakes may be highly sensitive to the ongoing changes and must be protected.

## Introduction

An accurate knowledge of species geographic distributions is critical to preserve biodiversity in a changing world. Despite the increasing availability of digitized information on biodiversity data and species occurrence, our knowledge about the geographic distribution of most species is regretfully incomplete [1–3]. This shortcoming, called the Wallacean shortfall, is more evident in highly diverse tropical ecosystems [4–6], which are paradoxically the most threatened ecosystems [7,8]. Moreover, the lack of data on species distribution is frequently biased toward small, inconspicuous, and not easily detectable species [1,9]. Even with the wide use of methodological tools to predict species ranges, large gaps still exist for these species, particularly because the primary data used in predictive models are scarce or incomplete, precluding a clear understanding of the threats from climate change and assessments of their conservation status.

Ongoing climate changes have already impacted organisms’ distributions around the world in recent years [10–13]. Snakes are especially sensitive to climate changes [14–16] because most species maintain daily activities in a restricted range of temperatures [17] and because the reproductive strategies in tropical species depend on rainfall patterns and seasonality in precipitation [17–20]. The most important issue regarding the impact of climate change is that species that are unable to evolve rapid physiological adaptations or climatic tolerances (to new thermal conditions) may become extinct unless they have good dispersal abilities to track suitable habitats [21–24]. These impacts are particularly harmful for those species that are habitat specialists, which would probably fail to migrate following suitable climates [23,25]. Despite this situation, with few exceptions, snakes have been neglected from studies assessing the impacts of climate change [24,26,27].

*Micrurus lemniscatus* Linnaeus 1758 comprises a taxonomic complex of six taxa: *M*. *l*. *lemniscatus*, *M*. *l*. *carvalhoi*, *M*. *diutius*, *M*. *l*. *helleri*, *M*. *potyguara* and *M*. *serranus*, which occur all across South America, except Chile and Uruguay. Despite the wide geographic distribution (encompassing such distinct biomes as the Amazon, Cerrado, Caatinga and Atlantic Forest) and medical importance due to their highly neurotoxic venoms, little is known about their ecology, taxonomic delimitation, or geographic distribution [28–32]. Some studies suggested that the *M*. *lemniscatus* complex is composed of two geographically distinct clades: one from Amazonia and another from the Caatinga and Atlantic forest biomes [29]. However, the limited number of individuals analyzed precluded a clear geographic delimitation of each form. Extensive compilations such as by Campbell & Lamar [33]reproduced these shortcomings and presented poor definitions of the species’ geographic limits, which were based on previous studies without covering the entire scope of information from the specimens available in scientific collections. Thus, this group clearly exemplifies the necessity for new studies dealing with the Wallacean shortfall, as the knowledge about species distributions is very incomplete. Most importantly, similar to the observations for other coralsnakes [30,34,35], the high levels of habitat degradation in the biomes where they occur may represent a severe threat for their long-term survival. This degradation raises the point that we may be losing these species even before they are fully known.

In this paper, we address the Wallacean shortfalls for the *M*. *lemniscatus* complex by refining our knowledge about the geographic distribution of species based on ecological niche modeling tools and a set of thoroughly examined occurrence records from scientific collections. From the present ranges, we project the distributions into future scenarios of climate change and address the potential impacts for the long-term conservation of these species. Finally, we evaluate the efficiency of the current reserve network in maintaining suitable areas for the species in the present and future. Our findings add valuable new information to the geographic distribution of each taxon, pointing to the necessity of reconsidering the conservation efforts for these less conspicuous species at risk of extinction in the near future.

## Materials and methods

### Species and climate data

Occurrence records were obtained from the scrutiny of all specimens belonging to the *M*. *lemniscatus* complex deposited in 33 museum collections in South America, the United States of America and Europe (see S1 Table and S1 Appendix for the list of specimens and the museum collections accessed). The specimens were carefully examined to confirm taxonomic identifications and avoid nomenclatural and georeferencing errors. A total of 768 occurrence records were mapped into a geographic grid encompassing South America, with cell boundaries following 0.5 × 0.5 degrees of latitude and longitude (the resolution of the climate data used for niche modeling, see below). Repeated records within a grid cell were excluded, thus reducing the spatial aggregation of occurrences and avoiding spatial autocorrelation effects in ecological niche modeling. The final number of records (unique records in each grid cell) was 75 for *M*. *l*. *lemniscatus*, 97 for *M*. *l*. *carvalhoi*, 33 for *M*. *diutius*, 49 for *M*. *l*. *helleri*, and three for *M*. *potyguara* (S1 Fig and S2 Table). Because there are problems in modeling data with few observations, we excluded *M*. *potyguara* from the analyses. We also did not use the data related to *M*. *serranus* owing to its unstable taxonomic position within the *M*. *lemniscatus* complex.

Because a clear spatial aggregation of records still remained for *M*. *l*. *carvalhoi*, probably representing a sampling bias in a highly populated region (S1 Fig), we applied the protocol proposed by Oliveira et al. [36] to reduce spatial aggregation and select geographically equidistant points for this species. We then compare the results obtained from using all records at 0.5°x0.5° resolution with the results after controlling for spatial aggregation. First, we calculated the Mahalanobis distances (D^2^) in environmental space, which was formed by the five bioclimatic variables used to build ecological niche models (ENMs, see below), between each one occurrence records and their centroid. Second, with the distances D^2^ and the geographical coordinates (i.e., latitude and longitude values) we fitted a simultaneous autoregressive (SAR) model to find the autoregressive coefficient *p* which measures the amount of spatial autocorrelation in data. Third, this coefficient *p* was used to calculate the effective degrees of freedom using the formula provided by Griffith [37]. Thus, the degrees of freedom were the effective number of independent records (presences and pseudo-absences). Finally, with the number of independent records (i.e., 60 points) we select in the environmental space the most equidistant ones using an algorithm that iteratively searches records which are most distant from each other. Since no considerable differences were observed between the results using all records (i.e., 97 points) and those after selecting equidistant records (i.e. 60 points) (S2 Fig and S2 Appendix), we presented here only the results using all records at 0.5°x0.5° resolution.

A set of bioclimatic variables at 0.5° resolution were obtained from the ecoClimate database (http://ecoclimate.org, [38]). EcoClimate provides updated climatic simulations for several time periods (past, present, and future) derived from the Coupled Model Intercomparison Project–Phase5 (CMIP5) and Paleoclimate Modeling Intercomparison Project–Phase3 (PMIP3) [39,40]. Given the variety of climatic simulations (as well as ecological niche models (ENMs hereafter), see below) currently available, we combined the output predictions from different climate models and ENMs following the ensemble approach of Araújo & New [41] (see details below). Thus, we used five coupled atmosphere-ocean general circulation models (AOGCMs: CCSM, CNRM, GISS, MIROC, and MRI) (S3 Table), with simulations for the present (represented by preindustrial data) and future (represented by mean values between 2080–2100), to derive 19 bioclimatic variables according to Hijmans et al. [42]. For the future, we used the emission scenario RCP4.5, which is an intermediate scenario between the lower (RCP2.6) and the higher (RCP8.0) emission scenarios [40].

To reduce collinearity between the bioclimatic variables in the ENMs, we applied a varimax-rotated factor analysis to the correlation matrix between pairs of variables and selected the variable with the highest loading in each one of the first five rotated factors (S4 Table). The selected variables were the mean annual temperature, annual temperature range, precipitation of the wettest month, precipitation of the driest month, and precipitation of the warmest quarter. Because the bioclimatic variables were highly correlated across the AOGCMs, we performed the factor analysis only with the AOGCM CCSM and then applied the selection for all other AOGCMs.

### Ecological niche models and geographic distribution

The variety of alternative methods to model species niches have promoted a range of discussions about the individual performance of each method to estimate potential geographic distributions [43–45], although no agreement was reached regarding which is the best choice for particular aims. We opted to apply the ensemble forecasting approach [41,46,47] to generate consensus predictions about geographic distributions after combining the outputs from several different methods.

We used 12 ENMs, including presence-only, presence-background and presence-absence methods, ranging from simple bioclimatic envelope models (e.g., BIOCLIM) and distance-based methods (e.g., Euclidian distance) up to more complex regression models such as (GLM) and machine learning-based methods (artificial neural networks) [48,49] (S5 Table). Models were built for the present using preindustrial climate data and projected into climate scenarios for the end of this century (mean simulations between 2080 and 2100).

The area used to adjust and project ENMs must correspond to regions that have been available for colonization to the species over relevant time periods [50]. The *Micrurus* genus is predominantly Neotropical, with more than 30 species occurring through the South America (the richest region for this clade) and only one species occurring in extreme south of North America [51]. Also, the taxa belonging to the *M*. *lemniscatus* complex are sibling species that were already considered as a single species distributed across most of the South American continent. Thus, we considered South America as the suitable area for model calibration and projection for this group.

For the modeling processes, we randomly divided species presence records (and pseudo-absences for those methods that use absences) into 75% for calibration and 25% for evaluation and repeated this process 50 times. Pseudo-absences were randomly selected in background regions (excluding cells with occurrence records) with the same proportion of species records (a prevalence of 0.5). The 50 repetitions in each method were converted in presence-absence maps according to thresholds established by the area under the ROC curve (AUC). All 600 models (50 repetitions × 12 modeling methods) were included in the consensus maps of each AOGCM, weighted by their model fit according to the true skill statistics–TSS [52] (S6 Table). The average across the ensemble outputs of each AOGCM resulted in the final consensus map of habitat suitability (varying from 0 to 1) for each species (S1 Fig). All models were generated using the computational platform BioEnsembles [46,47].

The agreement among projected distributions from the different ENMs and AOGCMs was assessed through a hierarchical ANOVA [46,47]. For this, the suitability of each cell from each combination of ENM and AOGCM was the response variable replicated (or nested) within the “time” component (present and future projections). We then used the sum of squares (SS) that can be attributed to each of these sources of variation (i.e., among ENMs and AOGCMs, both nested in time, and between the two time periods) to evaluate the uncertainties in species distribution. Low values of SS from ENMs and AOGCMs indicate high consistency in predictions from different methods and climate models. High values of SS from the time component indicate changes in suitability from the present to the future.

We used habitat suitability predictions to map the potential geographic distribution of each species in both time periods (present and future). Because ENMs do not consider biotic interactions and other local factors limiting species ranges (e.g., geographic barriers), their transferability (extrapolation in geographic space) may include areas that are inaccessible for the species. As we are dealing with poor dispersal species, we should be conservative to delineate more realistic and interpretable distributions. Thus, we applied the decision threshold based on the lowest presence threshold (LPT) [53], selecting the lower suitability value associated with the observed occurrence records. Following this, an area (or cell) was considered suitable if it had a certain value of suitability higher than the threshold. The final maps were compared with the expert opinions (N.J.S.Jr. and D.T.) to ensure that the predictions reflect the real potential distribution of each species.

Finally, we explored the relative importance of each bioclimatic variable used in niche models in determining the habitat suitability for each species by adjusting a multiple regression model between suitability and the bioclimatic variables. We then interpreted the standardized regression coefficients to set the contribution of each variable. Although the significance of these coefficients cannot be considered due to the implicit collinearity between suitability and the bioclimatic variables, this approach was useful for describing their relative contributions to the delimitation of species’ geographic distributions and to infer how climate changes could impact species in the future.

### Climate change and species representation in protected areas

We evaluated the potential impacts of future climate change by comparing the number of currently occupied cells in relation to future distributions. We also performed a gap analysis [54–57] by measuring the level of representation of species distributions into the protected areas according to habitat suitability for the present and future. To delineate the geographic extension of this analysis for each species, we overlapped the predicted geographic distributions (after cutting by specific LPT thresholds) with the South American ecoregions obtained from The Nature Conservancy GIS database (http://maps.tnc.org/about.html). We then selected the ecoregions that intersect with the species distributions using QGIS v. 2.12 (https://www.qgis.org/pt_BR/site/) (S3 Fig) and calculated the proportion of protected areas in each cell grid overlapping the selected ecoregions. The spatial distribution of the protected areas was obtained from the World Database on Protected Areas of the IUCN (available at: http://www.protectedplanet.net/search).

From the proportion of each cell that is currently protected and the continuous values of habitat suitability, we applied the species representation index *SRI* proposed by Alagador et al. [57]to evaluate how the species suitability is distributed in relation to the protected areas in the present and how it would be in the future by considering climate change. We also assessed the degree to which reserve networks represent species better than expected by chance alone. For this assessment, we randomly selected through 1,000 permutations an equal number of cells as the number of protected grid cells and redistributed the suitability values within the grid cells. We then compared the *SRI* value obtained for each species using the real reserve network with those obtained using the corresponding random set of reserves. We applied two-tailed tests by counting the results in which the *SRI* value was lower or higher than the real *SRI*. In each case, we checked whether the real *SRI* was greater than the 95^th^ or lower than 5^th^ percentile of the *SRI* values obtained from the randomizations. The *SRI* index has the advantage of using continuous data regarding species distributions (habitat suitability) and reserve coverage (proportion of the reserve area by grid cell), thus reducing the uncertainty in choosing arbitrary thresholds to convert continuous suitability into species presence and absence and to convert a grid cell into being considered either protected or unprotected [57].

## Results

### Niche models and species geographic distributions

**Fig 1:**
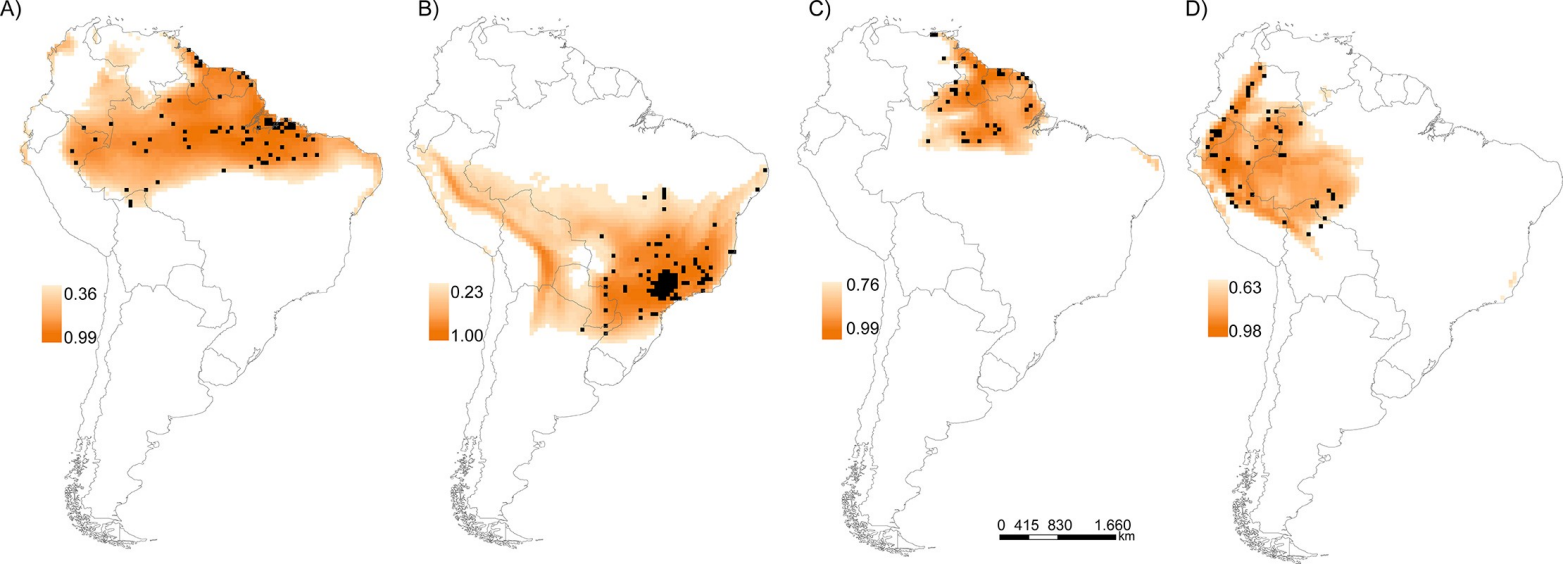
Habitat suitability and current geographic distribution. Consensus patterns of habitat suitability and potential present geographic distribution of the *Micrurus lemniscatus* species complex after applying specific decision thresholds (the lowest presence threshold). Black dots indicate occurrence records used in the models; A) *Micrurus l*. *lemniscatus*, B) *Micrurus l*. *carvalhoi*, C) *Micrurus diutius*, D) *Micrurus l*. *helleri*.

The ecological niche models exhibited good predictive performance (S6 Table), with mean TSS values across the AOGCMs and ENMs higher than 0.5 for all species except *M*. *l*. *helleri* (TSS = 0.45). The current distribution of each species after applying the LPT threshold (Table 1) was compatible with the known distribution from the observation records (Fig 1) and the expert opinions, except for *M*. *l*. *carvalhoi* (see below). Analyses of the uncertainties indicated that the climate models represented the lowest source of variation among the predictions (Table 2). Niche models were the second highest source of variation for two subspecies (*M*. *l*. *lemniscatus* and *M*. *l*. *helleri*), and the differences through present and future predictions were higher than those for the other two taxa (*M*. *l*. *carvalhoi* and *M*. *diutius*) (S4 Fig).

**Table 1:** Changes in geographic distribution measured as the difference in the number of 0.5 × 0.5° cells in which species are predicted to occur in the present and in the future. LPT: Lowest presence threshold used to cut the potential distributions.

Species	*Range size_P_	*Range size_F_	% of loss/gain	LTP
*M*. *l*. *lemniscatus*	1958	2364	20.7	0.36
*M*. *l*. *carvalhoi*	2292	1536	-32.9	0.23
*M*. *diutius*	616	82	-86.6	0.72
*M*. *l*. *helleri*	1066	600	-43.7	0.63

* Number of cells; P–present; F–future.

**Table 2:** Summary of the hierarchical ANOVA output where species environmental suitability was used as response variable, and atmosphere-ocean general circulation models (AOGCM) and ecological niche models (ENM) were used as categorical variables nested into two time periods (the present and the end of the century). The values represent the mean, maximum and minimum percentages of the sum of squares through all cells.

	*M*. *l*. *lemniscatus*		*M*. *l*. *carvalhoi*		*M*. *diutius*		*M*. *l*. *helleri*	
Variation source	Mean	Min.-Max.	Mean	Min.-Max.	Mean	Min.-Max.	Mean	Min.-Max.
**AOGCM [Time]**	5.75	0.00–48.84	4.80	0.00–59.80	3.49	0.00–60.43	2.68	0.00–31.85
**ENM [Time]**	25.29	1.22–84.73	20.30	1.55–89.54	25.12	0.80–82.27	34.09	1.67–90.39
**Time**	23.48	2.26–79.39	29.19	0.85–79.29	27.69	1.69–80.76	21.59	1.54–67.44
**Residual**	45.48	9.56–79.43	45.70	3.26–79.54	43.70	12.48–77.19	41.64	6.39–72.43

The niche models indicated that *Micrurus l*. *lemniscatus* is widely distributed across the Amazon basin in northern Brazil, extreme northern Peru, southern Colombia, in small regions southern and western Venezuela, and across the Guianas (Fig 1A). In Brazil, the *M*. *l*. *lemniscatus* distribution also extends towards the northern part of the Cerrado and Caatinga biomes. *Micrurus l*. *carvalhoi* also showed a broad distribution, extending from the Atlantic rainforest in southeastern Brazil, through the Cerrado biome towards eastern Paraguay, and Misiones and Corrientes in Argentina, including part of Bolivia and Peru, reaching the southern limits of the Amazon rainforest (Fig 1B). The distribution of *M*. *l*. *carvalhoi* also includes most of the northeastern region of Brazil. *Micrurus diutius* exhibited a restricted distribution in the rainforest of the Guianas and northern Brazil (Fig 1C). The predicted distribution for *M*. *l*. *helleri* was congruent with the recognized occurrence in the western region of the Amazon rainforest, including northern Bolivia and Brazil, northern and eastern Peru, eastern Ecuador and central and southern Colombia (Fig 1D).

Extreme precipitation conditions and precipitation during the warmest season, respectively represented by the variables Bio13 and Bio18, had the highest standardized coefficients for all species except *M*. *diutius* (Table 3). For this species, temperature variation was more important (Bio 7 –annual temperature range).

**Table 3:** Standardized regression coefficients from the multiple regression models between habitat suitability and the bioclimatic variables. Bold values indicate the two most important variables (i.e., with the higher coefficient values) for each species.

Species	Bio1	Bio7	Bio13	Bio14	Bio18
*M*. *l*. *lemniscatus*	**0.33**	-0.21	**0.45**	0.14	-0.24
*M*. *l*. *carvalhoi*	0.27	0.06	**-0.36**	-0.29	**0.47**
*M*. *diutius*	**0.27**	**-0.47**	0.21	0.12	-0.20
*M*. *l*. *helleri*	**0.40**	-0.30	-0.16	0.08	**0.41**

Bio1 –Annual Mean Temperature, Bio7 –Temperature Annual Range, Bio13 –Precipitation of Wettest Month, Bio14 –Precipitation of Driest Month, Bio18 –Precipitation of Warmest Quarter.

### Climate change and gap analysis

**Fig 2:**
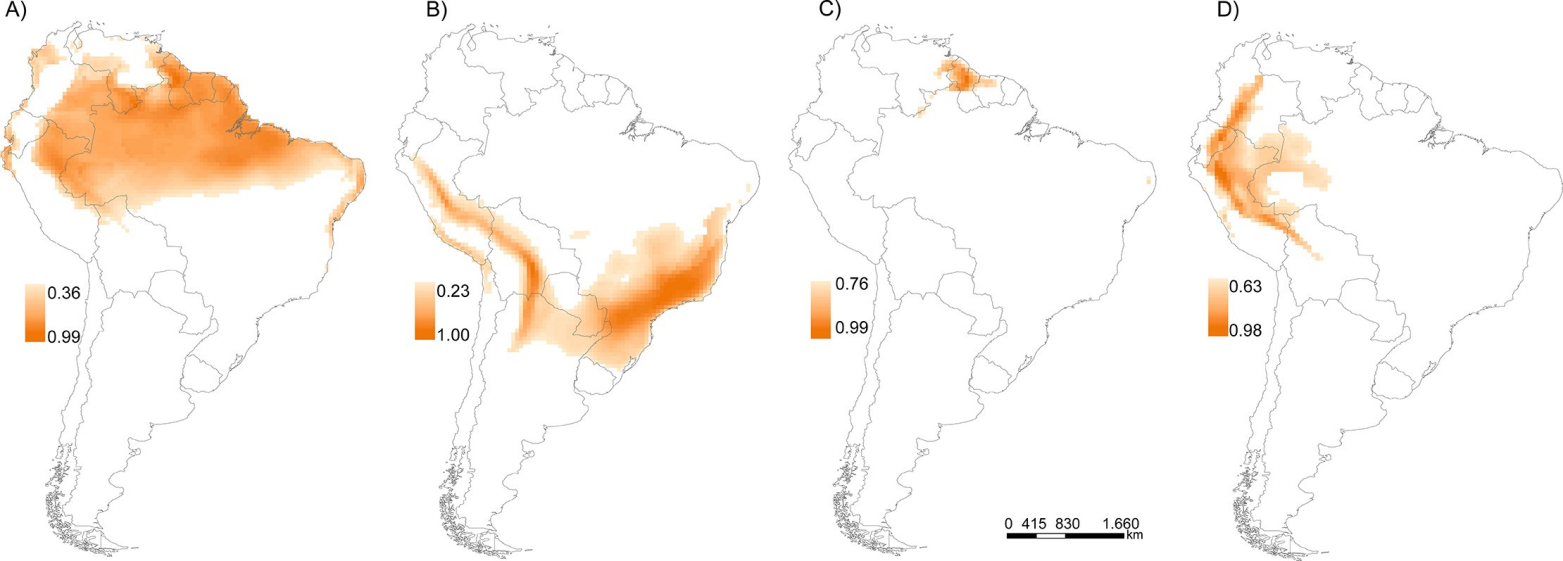
Habitat suitability and future geographic distribution. Consensus patterns of habitat suitability and potential future geographic distributions of the *Micrurus lemniscatus* complex after applying specific decision thresholds (the lowest presence threshold); A) *Micrurus l*. *lemniscatus*, B) *Micrurus l*. *carvalhoi*, C) *Micrurus diutius*, D) *Micrurus l*. *helleri*.

Except for *M*. *l*. *lemniscatus*, all species were projected to contract their distributions in the future (Fig 2). *Micrurus diutius* exhibited the most severe contraction (Fig 2C), with a reduction of 86% in its suitable area (Table 1). Substantial reductions were also projected for *M*. *l*. *carvalhoi* and *M*. *l*. *helleri*, with losses of 32 and 45%, respectively (Fig 2B and 2D).

**Fig 3:**
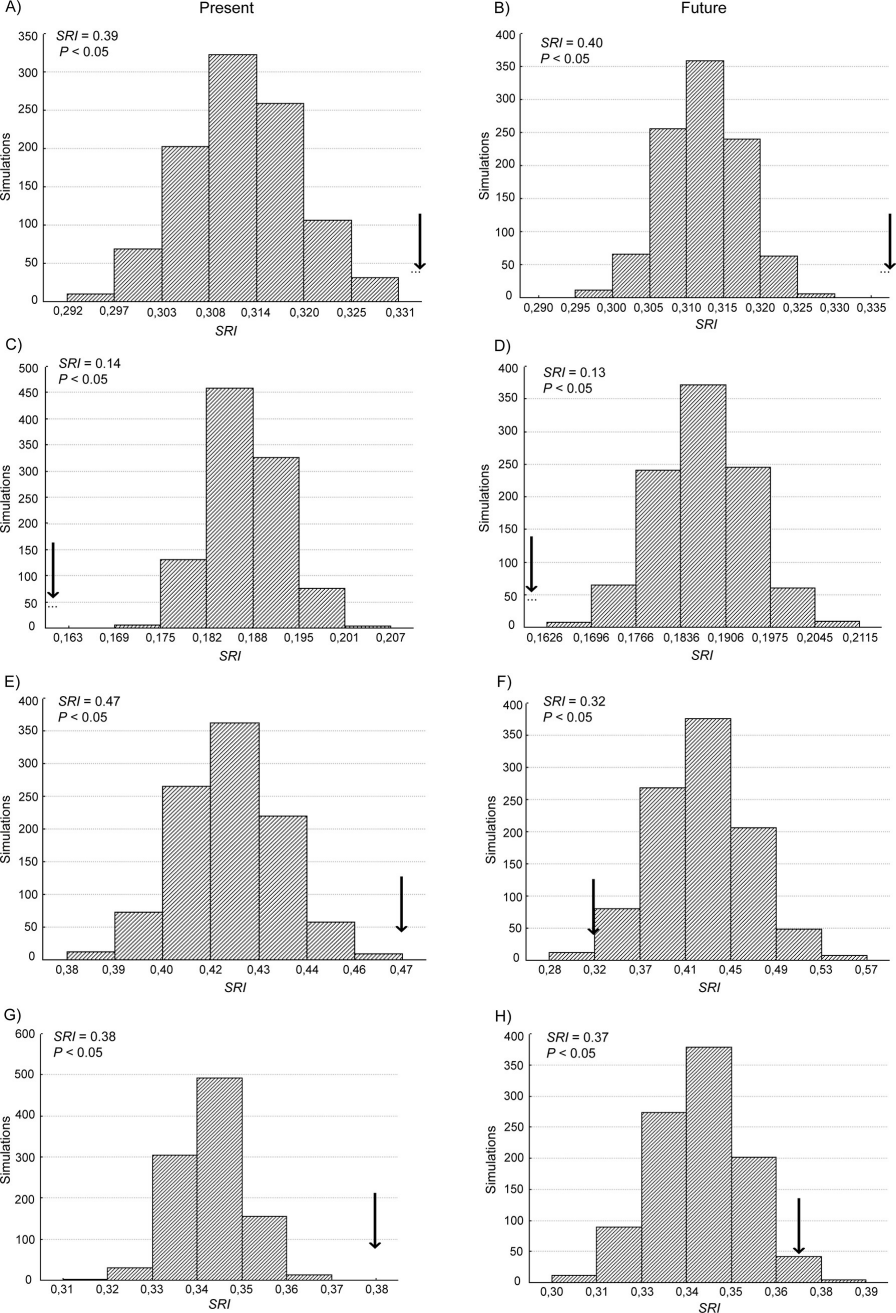
Species representation index (*SRI*). Frequency distributions of 1000 permutations of species representation index by randomly selecting protected areas within the ecoregions encompassed by each species distribution at present and in the future. The arrow indicates the position of the real *SRI* value relative to the distribution of randomized values for each species; significance at a two-tailed test is also indicated as *p*-values; A and B) *Micrurus l*. *lemniscatus*, C and D) *Micrurus l*. *carvalhoi*, E and F) *Micrurus diutius*, G and H) *Micrurus l*. *helleri*.

The *SRI* estimates based on continuous values of habitat suitability and the proportion of reserve coverage in the grid cells indicated that for all species, less than 50% of the present suitable areas are protected in reserve networks (Table 4). Even worse, all species will experience future suitability loss within the reserves, as less than 40% of the suitable areas are expected to be held in reserves at the end of the century. *M*. *l*. *carvalhoi* was the worst represented species in the protected areas (14% for the present and 13% for the future). The permutations (Fig 3) showed that for two subspecies (*M*. *l*. *lemniscatus* and *M*. *l*. *helleri*, Fig 3A, 3B, 3G and 3H), the current spatial configuration of the reserve network holds more suitable areas than those expected by their random distribution. However, for *M*. *l*. *carvalhoi* (Fig 3C and 3D), the network of reserves performed worse than expected to change, both for the present and future distributions. This poor performance means that the current distribution of the protected areas is insufficient to preserve *M*. *l*. *carvalhoi*, both at present and in the face future of climate changes. A similar result was observed for *M*. *diutius*, for which the loss of representation will result in a significant inadequacy of the reserves in the future (Fig 3F).

**Table 4:** Species representation index (*SRI*) and *p*-values from randomizations.

Species	*SRI*_present_	*SRI*_future_	*p*_present_	*p*_future_
***M*.** *l*. *lemniscatus*	0.39	0.40	< 0.05	< 0.05
***M***. *l*. *carvalhoi*	0.14	0.13	< 0.05	< 0.05
***M***. *diutius*	0.47	0.32	< 0.05	< 0.05
***M***. *l*. *helleri*	0.38	0.37	< 0.05	< 0.05

## Discussion

### Reducing the Wallacean shortfalls

Wallacean shortfall refers to our lack of knowledge of species distributions, both because we do not have adequate sampling efforts across multiple regions and lack straightforward methods to generalize from these data to obtain a clear picture of species distributions [1,2]. Data compilations and syntheses from biological collections, coupled with ENMs, have been widely used in the last 20 years as a way to reduce Wallacean shortfalls. In this sense, if the knowledge of biodiversity improves in the data and theory/models, it is possible to better understand the potential impacts of climate changes on species' geographic ranges [58,59]. Here, we used this approach to generate robust predictions of the geographic ranges for the *M*. *lemniscatus* species complex, thus contributing to the guidance nonrandom field surveys and to the evaluation of the shifts in their distributions in response to climate change.

Regarding the current distribution estimated for each species, the potential distribution of *M*. *l*. *lemniscatus* is in agreement with the recent proposition of Silva Jr. et al. [31] and extends the distribution originally proposed by Campbell & Lamar [33] from a small area in northern Brazil to a wide area encompassing most of the Amazon biome. The map of habitat suitability indicates that potential areas for collection efforts are located in the western Amazonas and the Acre Brazilian states, south of Colombia and west of Venezuela. However, the distribution of *M*. *l*. *carvalhoi* largely differs from that suggested by Campbell & Lamar [33] and covers a larger area than that proposed by Silva Jr. et al. [31]. However, the wide area predicted by ENMs covering northern Argentina, through Bolivia, Peru and southwestern Amazonas must be considered with caution, given that no occurrence record was collected in these regions. These areas have low suitability values (S1 Fig), as indicated by the lowest presence threshold (0.23), probably as an effect of the high aggregation of occurrence records in the distribution core, thus resulting in lower suitability values in the distribution border where some occurrence records are placed. Despite this potential methodological artifact, our results clearly suggest that *M*. *l*. *carvalhoi* is allopatric with the Amazonian *lemniscatus*, probably indicating the evolution of distinct habitat preferences (humid forests in the case of *lemniscatus* and open and dry forests in the case of *carvalhoi*) [29].

The distribution of *Micrurus diutius* was slightly expanded compared to previous studies [31,33], and a gap in the occurrence records in the northern border between Brazil, the Guianas and Suriname was suggested as an important region for further field surveys. *M*. *diutius* was recently reconsidered as a full species [60], but this status still warrants investigation. As *M*. *diutius* is sympatric with *M*. *l*. *lemniscatus* in most parts of its distribution, additional records from the undersampled but highly suitable areas could provide more accurate information to support splitting both species.

The distribution of *M*. *l*. *helleri* was more restricted than that proposed by Campbell & Lamar [33], and a larger area in northwestern Amazonia was indicated as having high suitability despite being undersampled. However, the status of this subspecies was recently reviewed by Silva Jr. et al. [31], who proposed its synonymy with *M*. *l*. *lemniscatus*. If so, the distribution of *M*. *l lemniscatus* will extend through most of Peru and Ecuador and probably reach the western and northern borders of the *M*. *l*. *carvalhoi* distribution.

### Climate change and gap analysis

Studies showing the impacts of climate change are common for vertebrate species worldwide [58,61–65], but only a few of those studies have specifically addressed the potential effects on snakes [16,24,66,67]. These few studies provide clear evidence that snakes are very sensitive to climate changes, with some species undergoing severe range shifts [66,67], range contractions [68], or drastic population declines [16]. As the first study about the impacts of climate change on coralsnakes, we report here the alarming evidence that for three out the four species analyzed, the geographic distributions will probably shrink in the future.

Although more detailed (including experimental) studies are necessary to assess the susceptibility of coralsnakes to climate change, we can infer the potential impacts based on specific traits that make species more sensitive to climate change, as summarized by Foden et al. [69]. First, coralsnakes have specialized habitats and specific microhabitat requirements based on their semi-fossorial habit. For instance, drier soils caused by warmer temperatures and reduced precipitation may force these species to burrow deeper into the soil to find sufficient moisture or to hunt other fossorial prey [70]. If changes in soil humidity cause changes in the populations of other fossorial prey species such as amphisbaenians and caecilians, coralsnakes will probably also be affected because overall, snakes respond very strongly to prey densities [30,70,71].

Second, their physiology and ecology is strongly dependent on the specific ranges of climatic variables, such as temperature for thermoregulation, and precipitation and soil humidity for breeding cycles and foraging activities [30]. For instance, Marques et al. [17] showed that in triadal *Micrurus*, female ovulation occurs in spring, and males have a peak of spermatogenesis in the summer, synchronous with mating that occurs during this hot and humid season. Other similar studies corroborate the need for specific climatic conditions for the reproductive cycles of coralsnakes [30], and our exploratory analyses of the association between climate and habitat suitability confirms the importance of seasonal conditions (mainly precipitation) for these species. Therefore, rapid climate changes in the future probably will also require rapid evolutionary changes in phenology, which is rare (but see Moreno-Rueda et al. [68] and highly unlikely to occur in snakes [24].

Third, coralsnakes are also dependent on interspecific interactions, particularly regarding their diet specialization, consisting predominantly of amphisbaenians, caecilians and other snakes. Considering that *Micrurus* species are morphologically and ecologically conservative [29,30,33], and due to their high degree of diet specialization, they are unlikely to be able to switch to or substitute other food resources, increasing their susceptibility to disruption by climate changes.

And fourth, their poor ability to disperse to new suitable habitats because of their sedentary and subterraneous habitat prevents the species from tracking climate changes, which can be even worse due to the geographic barriers, given that some of these species are unable to overcome small rivers [30]. Thus, this negative combination of susceptible traits along with the climatically imposed reductions in suitable habitats is likely to highly threaten species in the future.

*Micrurus l*. *lemniscatus* was the only taxon for which future predictions indicate a gain in suitable areas (measured as the number of 0.5° cells) at the end of century, although part of this will occur in the western border of Peru, Ecuador and Colombia, i.e., outside the core of the distribution, where it is very unlikely for the species to colonize. In any case, a substantial increase in the suitable area was observed south of the current distribution (i.e., advancing towards the center of Brazil), and in central Colombia and southern Venezuela. As this species is dominant throughout the Amazon and is probably well adapted to the high temperatures and humidity characteristic of this biome [31,33,72], one could infer that it will be less affected by climate changes. However, we noted that as *M*. *l*. *lemniscatus* and *M*. *l*. *carvalhoi* are allopatric, the advance of the *M*. *l*. *lemniscatus* limits towards the *M*. *l*. *carvalhoi* distribution indicate the potential for novel interactions in the future, which can impose additional negative effects (e.g., competitive exclusion) on both species. Additional studies are necessary to evaluate the potential impacts of novel patterns of co-occurrence for these snakes, but the evidence provided here cannot be ignored.

Our study also indicates that for *M*. *l*. *carvalhoi*, *M*. *diutius* and *M*. *l*. *helleri*, the suitable habitats are poorly represented in the protected areas, both at present and for the future. This finding is particularly alarming for *M*. *l*. *carvalhoi*, which now has only 14% representation and 13% for the future, and whose distribution includes two highly threatened biomes—the Atlantic forest and the Cerrado. The Atlantic forest currently has only ca. 11% of its natural cover [73], and only ca. 7.2% of its remaining habitats are protected [74]. The Cerrado biome still retains ca. 45% of its natural cover [75], but its increasing transformation into pasture and cash-crop agriculture is noticeably rapidly reducing the natural areas. Even in the Amazon biome that still remains largely intact due to its great size, some species may be severely threatened. Thus, if the levels of habitat protection in the current reserve network are not sufficient, and species are being driven out of reserves due to climate change as we found here [76,77], the remaining intact vegetation must be protected, and new areas in these biomes should be the focus of intense research and conservation actions. Most importantly, more attention should be paid to those areas considered as long-term climatic refugia [47,78,79], since they can function as buffers to the impacts of climate changes and provide a valuable alternative to preserve *in situ* species with poor dispersal abilities (i.e., without the need for migration or translocation).

*Micrurus diutius* was predicted to lose more than 80% of suitable area. In this case, it is important to note that the suitability value used as a threshold (defined from the LPT) was quite high (i.e., 0.72), resulting in a reduced distributional area. Use of the LPT, although appropriate in cases where a more conservative prediction is desired, can be influenced by the number of locality records, which increases as the sample size decreases [53]. *Micrurus diutius* had the lowest number of occurrence records (only 33 spatially unique records at the resolution of 0.5°), which can explain the high value of the LPT. Thus, the interpretation of the high decrease in its distributional area due to climate change, and consequently, its low representation in the current network of protected areas, should be made with caution. Nevertheless, the present distributional area obtained after applying the LPT provides valuable clues for identifying new sites that are at least as suitable as those where the species has been recorded.

In summary, through the application of niche model tools and carefully revised occurrence records, we were able to reduce, with a good deal of certainty, the Wallacean shortfall for the *M*. *lemniscatus* species complex. For the first time, we report the potential impacts of climate changes on the distribution of coralsnakes, with strong evidence that these organisms may be highly sensitive to the ongoing changes. Coralsnakes, similar to other snake species, are middle-order predators, and therefore, their extinction may have serious consequences for the functioning of ecosystems [80]. Thus, we undoubtedly need exhaustive studies focused on the unexplored aspects of ecology and evolution of coralsnakes, which might have far-reaching implications for understanding their responses to the changing climate and supporting more effective conservation management.

## Supporting information

S1 Appendix List of museums. List of museums from which specimens were examined.
(PDF)

S2 Appendix Results of ENM for *M*. *l*. *carvalhoi*. Results of ENM and analyses for *M*. *l*. *carvalhoi* after controlling for spatial aggregation in occurrence records.
(PDF)

S1 Table List of occurrence records. List of occurrence records with the collection IDs. Collection abbreviations correspond to the museums listed in the S1 Appendix above.
(PDF)

S2 Table Number of occurrence records. Number of occurrence records from museum collections and after mapping over the South American grid.
(PDF)

S3 Table Climatic models. Details of the climatic simulations (AOGCMs) used in the ecological niche modeling.
(PDF)

S4 Table Output for factorial analysis. Loadings of the bioclimatic variables in the first five axes of varimax rotated factor analysis, based on the CCSM AOGCM. Numbers in bold highlight the highest loading of the selected variable in each factor.
(PDF)

S5 Table Ecological niche modeling methods. Ecological niche modeling methods used to estimate species potential distributions.
(PDF)

S6 Table Output of model evalution. The mean true skill statistics (TSS) values across five AOGCMs (CCSM, CNRM, GISS, MIROC and MRI) for each ENM method.
(PDF)

S1 Fig Habitat suitability maps. Consensus maps of habitat suitability derived from 12 niche modeling methods and 5 climate models. Hot colors indicate high habitat suitability; cool colors, low habitat suitability. Black dots indicate presence records used in the modeling processes; A) *Micrurus l*. *lemniscatus*, B) *Micrurus l*. *carvalhoi*, C) *Micrurus diutius*, D) *Micrurus l*. *helleri*.
(PDF)

S2 Fig Habitat suitability map for *M*. *l*. *cavalhoi*. Consensus maps of habitat suitability from ENMs for *M*. *l*. *cavalhoi* for A) present, and B) future after controlling for spatial autocorrelation in occurrence records. Black dots are the most equidistant records select in the environmental space.
(PDF)

S3 Fig Terrestrial ecoregions. Delimitation of the terrestrial ecoregions used to assess the level of species representation in protected areas. For each taxon, the selection of these areas was done by overlapping the map of habitat suitability (after applying specific decision thresholds) with the map of South American ecoregions. An ecoregion was selected if at least one cell overlapping it was predicted as present; A) *Micrurus l*. *lemniscatus*, B) *Micrurus l*. *carvalhoi*, C) *Micrurus diutius*, D) *Micrurus l*. *helleri*.
(PDF)

S4 Fig Spatial distribution of the sum of squares. Proportion of the sum of squares (SS) from the ANOVA accounted for by the ecological niche models (ENMs), atmosphere-ocean general circulation models (AOGCMs) and time (the present and the end of the century). Dark red indicates high SS; light red, low SS. A) *Micrurus l*. *lemniscatus*, B) *Micrurus l*. *carvalhoi*, C) *Micrurus diutius*, D) *Micrurus l*. *helleri*.
(PDF)

## References

[1] Hortal J, de Bello F, Diniz-Filho JAF, Lewinsohn TM, Lobo JM, Ladle RJ. Seven shortfalls that beset large-scale knowledge of biodiversity. Annu Rev Ecol Evol Syst. 2015;46: 523–549. 10.1146/annurev-ecolsys-112414-054400

[2] Whittaker RJ, Araujo MB, Jepson P., Ladle RJ, Watson JEM, Willis KJ. Conservation biogeography: asessment and prospect. Divers Distrib. 2005;11: 3–23. 10.1111/j.1366-9516.2005.00143.x

[3] Lomolino M V. Frontiers of Biogeography In: Lomolino M, Heaney L, editors. Frontiers of Biogeography: New Directions in the Geography of Nature. Sunderland, MA: Sinauer; 2004.

[4] Bush MB, Lovejoy TE. Amazonian conservation: Pushing the limits of biogeographical knowledge. J Biogeogr. 2007;34: 1291–1293. 10.1111/j.1365-2699.2007.01758.x

[5] Hopkins MJG. Modelling the known and unknown plant biodiversity of the Amazon Basin. J Biogeogr. 2007;34: 1400–1411. 10.1111/j.1365-2699.2007.01737.x

[6] Meyer C, Kreft H, Guralnick R, Jetz W. of biodiversity distributions. Nat Commun. Nature Publishing Group; 2015;6: 1–8. 10.1038/ncomms9221 26348291 10.1038/ncomms9221 PMC4569846

[7] Myers N, Mittermeier R a, Mittermeier CG, da Fonseca G a, Kent J. Biodiversity hotspots for conservation priorities. Nature. 2000;403: 853–8. 10.1038/35002501 10706275 10.1038/35002501

[8] Mittermeier RA, Gil PR, Hoffmann M, Pilgrim J, Brooks T, Mittermeier CG, et al Hotspots revisited University of Chicago Press, Boston, USA Boston, USA: University of Chicago Press; 2004 10.1017/CBO9781107415324.004

[9] Gaston KJ, Blackburn TM. Are newly described bird species small-bodied? Biodivers Lett. 1994;2: 16–20.

[10] Parmesan C, Yohe G. A globally coherent fingerprint of climate change impacts across natural systems. Nature. 2003;421: 37–42. 10.1038/nature01286 12511946 10.1038/nature01286

[11] Thomas CD, Cameron A, Green RE, Bakkenes M, Beaumont LJ, Collingham YC, et al Extinction risk from climate change. Nature. 2004;427: 145–8. 10.1038/nature02121 14712274 10.1038/nature02121

[12] IPCC. Climate Change 2014: Synthesis Report. 2014. pp. 1–151.

[13] Chen I. Rapid range shifts of species associated with high levels of climate warming. Science (80-). 2011;333: 1024–1026. 10.1126/science.1206432 21852500 10.1126/science.1206432

[14] Gibbons JW, Scott DE, Ryan T, Buhlmann JKA, Tuberville TD, Metts BS, et al of Reptiles, Déjà Vu Amphibians. Bioscience. 2000;50: 653–666.

[15] Aubret F, Shine R. Thermal plasticity in young snakes: how will climate change affect the thermoregulatory tactics of ectotherms? J Exp Biol. 2010;213: 242–248. 10.1242/jeb.035931 20038657 10.1242/jeb.035931

[16] Reading CJ, Luiselli LM, Akani GC, Bonnet X, Amori G, Ballouard JM, et al Are snake populations in widespread decline? Biol Lett. 2010;6: 777–80. 10.1098/rsbl.2010.0373 20534600 10.1098/rsbl.2010.0373 PMC3001371

[17] Marques OAV, Pizzatto L, Santos SMA. Reproductive Strategies of New World Coral Snakes, Genus Micrurus. Herpetologica. 2013;69: 58–66. Available: http://www.hljournals.org/doi/abs/10.1655/HERPETOLOGICA-D-12-00091

[18] Marques OA V. Reproduction, seasonal activity and growth of the coral snake, Micrurus corallinus (Elapidae), in the southeastern Atlantic forest in Brazil. Amphibia-Reptilia. 1999;17: 277–285.

[19] Marques OAV. Natural history of the coral snake Micrurus decoratus (Elapidae) from the Atlantic Forest in southeast Brazil, with comments on possible mimicry [Internet]. Amphibia-Reptilia 2002 pp. 228–232. Available: http://www.ecoevo.com.br/publicacoes/pesquisadores/otavio_marques/2002_naturalhistorycoralsnakemdecoratus_2002.pdf

[20] Brown GP, Shine R. Rain, prey and predators: Climatically driven shifts in frog abundance modify reproductive allometry in a tropical snake. Oecologia. 2007;154: 361–368. 10.1007/s00442-007-0842-8 17724615 10.1007/s00442-007-0842-8

[21] Huey RB, Hertz PE, Sinervo B. Behavioral drive versus Behavioral inertia in evolution: A null model approach. Am Nat. 2003;161: 357–366. 10.1086/346135 12699218 10.1086/346135

[22] Diniz-Filho JAF, Bini LM. Macroecology, global change and the shadow of forgotten ancestors. Glob Ecol Biogeogr. 2007;17: 11–17. 10.1111/j.1466-8238.2007.00339.x

[23] Sinervo B, Mendez-de-la-Cruz F, Miles DB, Heulin B, Bastiaans E, Villagran-Santa Cruz M, et al Erosion of lizard diversity by climate change and altered thermal niches. Science (80-). 2010;328: 894–899. 10.1126/science.1184695 20466932 10.1126/science.1184695

[24] Lawing AM, Polly PD. Pleistocene climate, phylogeny, and climate envelope models: An integrative approach to better understand species’ response to climate change. PLoS One. 2011;6: e28554 10.1371/journal.pone.0028554 22164305 10.1371/journal.pone.0028554 PMC3229599

[25] Pincheira-Donoso D, Tregenza T, Witt MJ, Hodgson DJ. The evolution of viviparity opens opportunities for lizard radiation but drives it into a climatic cul-de-sac. Glob Ecol Biogeogr. 2013;22: 857–867. 10.1111/geb.12052

[26] Nori J, Carrasco PA, Leynaud GC. Venomous snakes and climate change: Ophidism as a dynamic problem. Clim Change. 2013;122: 67–80. 10.1007/s10584-013-1019-6

[27] Yañez-Arenas C, Peterson AT, Mokondoko P, Rojas-Soto O, Martínez-Meyer E. The use of ecological niche modeling to infer potential risk areas of snakebite in the Mexican State of Veracruz. PLoS One. 2014;9: e100957 10.1371/journal.pone.0100957 24963989 10.1371/journal.pone.0100957 PMC4071012

[28] Silva NJ Da Jr., Sites JW Jr.. Revision of the Micrurus frontalis complex (Serpentes: Elapidae). Herpetol Monogr. 1999;13: 142–194. 10.2307/1467062

[29] Silva NJ Da Jr., Sites JW Jr.. Phylogeny of South American triad coral snakes (Elapidae: Micrurus) based on molecular characters. Herpetologica. 2001;57: 1–22.

[30] Almeida PCR de, Prudente AL da C, Curcio FF, Rodrigues MTU. Biologia e História Natural das Cobras-Corais In: Silva NJ Jr., editor. As Cobras Corais do Brasil: Biologia, Taxonomia, Venenos e Envenenamentos. Goiânia: PUC Goiás; 2016 pp. 168–215.

[31] Silva NJ da Jr., Pires MG, Feitosa DT. Diversidade de Cobras Corais do Brasil In: Silva r. NJ da, editor. As Cobras Corais do Brasil: Biologia, Taxonomia, Venenos e Envenenamentos. Goiânia: PUC Goiás; 2016 pp. 78–167.

[32] Silva NJ da Jr., Buononato MA, Feitosa DT. As Cobras Corais do Novo Mundo In: Silva NJ da Jr, editor. As Cobras Corais do Brasil: Biologia, Taxonomia, Venenos e Envenenamentos. Goiânia: PUC Goiás; 2016 pp. 46–77.

[33] Campbell JA, Lamar WW. The Venomous Reptiles of the Western Hemisphere First Edit Ithaca: Cornell University Press; 2004.

[34] Silva NJ da Jr. Novas ocorrências de Micrurus brasiliensis Roze, 1967 (Serpentes: Elapidae) em áreas de tensão ambiental no centro-oeste brasileiro. Estudos. 2007;34: 931–956.

[35] Pires MG, Da Silva NJ, Feitosa DT, Da Costa Prudente AL, Pereira Filho GA, Zaher H. A new species of triadal coral snake of the genus Micrurus Wagler, 1824 (Serpentes: Elapidae) from northeastern Brazil. Zootaxa. 2014;3811: 569–584. doi: 10.11646/zootaxa.3811.4.8 24943187 10.11646/zootaxa.3811.4.8

[36] de Oliveira G, Rangel TF, Lima-Ribeiro MS, Terribile LC, Diniz-Filho JAF. Evaluating, partitioning, and mapping the spatial autocorrelation component in ecological niche modeling: A new approach based on environmentally equidistant records. Ecography (Cop). 2014;37: 637–647. 10.1111/j.1600-0587.2013.00564.x

[37] Griffith DA. Spatial autocorrelation and spatial filtering–gaining understanding through theory and scientific visualization Springer US; 2003.

[38] Lima-Ribeiro MS, Varela S, González-Hernández J, de Oliveira G, Diniz-Filho JAF, Terribile LC. Ecoclimate: a database of climate data from multiple models for past, present, and future for macroecologists and biogeographers. Biodivers Informatics. 2015;10: 1–21. doi: 10.17161/bi.v10i0.4955

[39] Braconnot P, Harrison SP, Kageyama M, Bartlein PJ, Masson-Delmotte V, Abe-Ouchi A, et al Evaluation of climate models using palaeoclimatic data. Nat Clim Chang. Nature Publishing Group; 2012;2: 417–424. 10.1038/nclimate1456

[40] Taylor KE, Stouffer RJ, Meehl GA. An overview of CMIP5 and the experiment design. Bull Am Meteorol Soc. 2012;93: 485–498. 10.1175/BAMS-D-11-00094.1

[41] Araújo MB, New M. Ensemble forecasting of species distributions. Trends Ecol Evol. 2007;22: 42–7. 10.1016/j.tree.2006.09.010 17011070 10.1016/j.tree.2006.09.010

[42] Hijmans RJ, Cameron SE, Parra JL, Jones PG, Jarvis A. Very high resolution interpolated climate surfaces for global land areas. Int J Climatol. 2005;25: 1965–1978. 10.1002/joc.1276

[43] Segurado P, Araújo MB. An evaluation of methods for modelling species distributions. J Biogeogr. 2004;31: 1555–1568.

[44] Elith J, Graham CH, Anderson RP, Dudík M, Ferrier S, Guisan A, et al Novel methods improve prediction of species’ distributions from occurrence data. Ecography (Cop). 2006;2: 129–151.

[45] Townsend Peterson A, Papeş M, Eaton M. Transferability and model evaluation in ecological niche modeling: a comparison of GARP and Maxent. Ecography (Cop). 2007;30: 550–560. 10.1111/j.2007.0906–7590.05102.x

[46] Diniz-Filho JAF, Mauricio Bini L, Fernando Rangel T, Loyola RD, Hof C, Nogués-Bravo D, et al Partitioning and mapping uncertainties in ensembles of forecasts of species turnover under climate change. Ecography (Cop). 2009;32: 897–906. 10.1111/j.1600-0587.2009.06196.x

[47] Terribile LC, Lima-ribeiro MS, Araújo MB, Bizão N, Collevatti RG, Dobrovolski R, et al Areas of climate stability of species ranges in the Brazilian Cerrado: disentangling uncertainties through time. Nat Conserv. 2012;10: 152–159.

[48] Peterson AT, Soberón J, Pearson RG, Anderson RP, Martinéz-Meyer E, Nakamura M, et al Ecological Niches and Geographic Distributions New Jersey: Princeton University Press; 2011.

[49] Franklin J. Mapping species distributions: spatial inference and prediction Cambridge: Cambridge University Press; 2009.

[50] Barve N, Barve V, Jiménez-Valverde A, Lira-Noriega A, Maher SP, Peterson AT, et al The crucial role of the accessible area in ecological niche modeling and species distribution modeling. Ecol Modell. Elsevier B.V.; 2011;222: 1810–1819. 10.1016/j.ecolmodel.2011.02.011

[51] Terribile LC, Olalla-Tárraga MA, Morales-Castilla I, Rueda M, Vidanes RM, Rodríguez MA, et al Global richness patterns of venomous snakes reveal contrasting influences of ecology and history in two different clades. Oecologia. 2009;159: 617–26. 10.1007/s00442-008-1244-2 19101733 10.1007/s00442-008-1244-2

[52] Allouche O, Tsoar A, Kadmon R. Assessing the accuracy of species distribution models: prevalence, kappa and the true skill statistic (TSS). J Appl Ecol. 2006;43: 1223–1232. 10.1111/j.1365-2664.2006.01214.x

[53] Pearson RG, Raxworthy CJ, Nakamura M, Townsend Peterson A. Predicting species distributions from small numbers of occurrence records: A test case using cryptic geckos in Madagascar. J Biogeogr. 2007;34: 102–117. 10.1111/j.1365-2699.2006.01594.x

[54] Scott JM, Davis F, Csuti B, Noss R, Butterfield B, Anderson H, et al Gap analysis: a geographic approach to protection of biological diversity. Wildl Monogr. 1993;123: 3–41.

[55] Araújo MB, Lobo JM, Moreno JC. The effectiveness of Iberian protected areas in conserving terrestrial biodiversity. Conserv Biol. 2007;21: 1423–1432. 10.1111/j.1523-1739.2007.00827.x 18173466 10.1111/j.1523-1739.2007.00827.x

[56] Rodrigues ASL, Akçakaya HR, Andelman SJ, Bakarr MI, Boitani L, Brooks TM, et al Global Gap Analysis: Priority Regions for Expanding the Global Protected-Area Network. Bioscience. 2004;54: 1092 10.1641/0006-3568(2004)054[1092:GGAPRF]2.0.CO;2

[57] Alagador D, Martins MJ, Cerdeira JO, Cabeza M, Araújo MB. A probability-based approach to match species with reserves when data are at different resolutions. Biol Conserv. Elsevier Ltd; 2011;144: 811–820. 10.1016/j.biocon.2010.11.011

[58] Araújo MB, Thuiller W, Pearson RG. Climate warming and the decline of amphibians and reptiles in Europe. J Biogeogr. 2006;33: 1712–1728. 10.1111/j.1365-2699.2006.01482.x

[59] Feeley KJ, Silman MR. The data void in modeling current and future distributions of tropical species. Glob Chang Biol. 2011;17: 626–630. 10.1111/j.1365-2486.2010.02239.x

[60] Starace F. Serpents et Amphisbénes de Guyane française Matoury: Ibis Rouge; 2013.

[61] Alford RA, Dixon PM, Pechmann JHK. Global amphibian population declines. 2001;412: 499–500. 10.1038/35087658 11484041 10.1038/35087658

[62] Both C, Bouwhuis S, Lessells C, Visser M. Climate change and population declines in a long-distance migratory bird. Nature. 2006;441: 81–84. 10.1038/nature04539 16672969 10.1038/nature04539

[63] Thuiller W, Broennimann O, Hughes G, Alkemade JRM, Midgley GF, Corsi F. Vulnerability of African mammals to anthropogenic climate change under conservative land transformation assumptions. Glob Chang Biol. 2006;12: 424–440. 10.1111/j.1365-2486.2006.01115.x

[64] Charmantier A, McCleery RH, Cole LR, Perrins C, Kruuk LEB, Sheldon BC. Adaptive phenotypic plasticity in response to climate change in a wild bird population. Science (80-). 2008;320: 800–803. 10.1126/science.1157174 18467590 10.1126/science.1157174

[65] Gouveia SF, Souza-Alves JP, Rattis L, Dobrovolski R, Jerusalinsky L, Beltrão-Mendes R, et al Climate and land use changes will degrade the configuration of the landscape for titi monkeys in eastern Brazil. Glob Chang Biol. 2016;22: 2003–2012. 10.1111/gcb.13162 26663738 10.1111/gcb.13162

[66] Moreno-Rueda G, Pleguezuelos JM, Alaminos E. Climate warming and activity period extension in the Mediterranean snake Malpolon monspessulanus. Clim Change. 2009;92: 235–242. 10.1007/s10584-008-9469-y

[67] Wu J. and Detecting attributing the effects of climate change on the distributions of snake species over the past 50 years. Environ Manage. Springer US; 2016;57: 207–219. 10.1007/s00267-015-0600-3 26289351 10.1007/s00267-015-0600-3

[68] Penman TD, Pike DA, Webb JK, Shine R. Predicting the impact of climate change on Australia’s most endangered snake, Hoplocephalus bungaroides. Divers Distrib. 2010;16: 109–118. 10.1111/j.1472-4642.2009.00619.x

[69] Foden W, Mace G, Vié J-C, Angulo A, Butchart S, Devantier L, et al Species susceptibility to climate change impacts. 2008 Rev IUCN Red List Threat Species. 2008; 1–12. 10.1128/AEM.01630-08

[70] Bickford D, Howard SD, Ng DJJ, Sheridan JA. Impacts of climate change on the amphibians and reptiles of Southeast Asia. Biodivers Conserv. 2010;19: 1043–1062. 10.1007/s10531-010-9782-4

[71] Shine R, Madsen T. Prey abundance and predator reproduction: rats and phytons on a tropical Australian floodplain. Ecology. 1997;78: 1078–1086.

[72] Roze JA. Coral Snakes of the Americas. Biology, Identification and Venoms. Malabar, Florida: Krieger Publishing Company; 1996.

[73] Ribeiro MC, Metzger JP, Martensen AC, Ponzoni FJ, Hirota MM. The Brazilian Atlantic Forest: How much is left, and how is the remaining forest distributed? Implications for conservation. Biol Conserv. Elsevier Ltd; 2009;142: 1141–1153. 10.1016/j.biocon.2009.02.021

[74] UNEP-WCMC. Data Standards for the World Database on Protected Areas UNEP-WCMC; 2010.

[75] Klink C a., Machado RB. Conservation of the Brazilian Cerrado. Conserv Biol. 2005;19: 707–713. 10.1111/j.1523-1739.2005.00702.x

[76] Araujo MB, Cabeza M, Thuiller W, Hannah L, Williams PH. Would climate change drive species out of reserves? An assessment of existing reserve-selection methods. Glob Chang Biol. 2004;10: 1618–1626. 10.1111/j.1365-2486.2004.00828.x

[77] Ferro VG, Lemes P, Melo AS, Loyola R. The reduced effectiveness of protected areas under climate change threatens atlantic forest tiger moths. PLoS One. 2014;9: e107792 10.1371/journal.pone.0107792 25229422 10.1371/journal.pone.0107792 PMC4168255

[78] Ashcroft MB. Identifying refugia from climate change. J Biogeogr. 2010;37: 1407–1413. 10.1111/j.1365-2699.2010.02300.x

[79] Werneck FP, Nogueira C, Colli GR, Sites JW, Costa GC. Climatic stability in the Brazilian Cerrado: Implications for biogeographical connections of South American savannas, species richness and conservation in a biodiversity hotspot. J Biogeogr. 2012;39: 1695–1706. 10.1111/j.1365-2699.2012.02715.x

[80] Petchey OL, McPhearson PT, Casey TM, Morin PJ. Environmental warming alters food-web structure and ecosystem function. Nature. 1999;402: 69–72. 10.1038/47023

