# Supplementary material for: Reducing Wallacean shortfalls for the coralsnakes of the Micrurus lemniscatus species complex: Present and future distributions under a changing climate
Source: PLoS One. 2018 Nov 14;13(11):e0205164. doi: 10.1371/journal.pone.0205164 (PMC6241113; doi:10.1371/journal.pone.0205164)
Supplement: S1 Appendix — List of museums from which specimens were examined. (PDF) [file pone.0205164.s001.pdf]

S1 Appendix. List of museums. List of museums from which specimens were examined.

Academy of Natural Sciences of Philadelphia, Philadelphia (ANSP); American Museum of Natural History, New York (AMNH); California Academy of Sciences, San Francisco (CAS); Carnegie Museum of Natural History, Pittsburgh (CM); Centro de Estudos e Pesquisas Biológicas da Pontifícia Universidade Católica de Goiás, Goiânia (CEPB); Coleção Zoológica da Universidade Federal de Mato Grosso do Sul, Campo Grande (ZUFMS); Coleção Zoológica Gregório Bondar, Ilhéus (CZGB); Departamento de Sistemática e Ecologia, Universidade Federal da Paraíba, João Pessoa (UFPB); Field Museum of Natural History, Chicago (FMNH); Fundação de Medicina Tropical, Manaus (FMT); Instituto de Ciencias Naturales de la Universidad Nacional de Colombia, Bogotá (ICN); Instituto de Investigación de Recursos Biológicos Alexander von Humboldt, Villa de Leyva (IAvH); Instituto Nacional de Pesquisas da Amazônia, Manaus (INPA); Instituto Butantan, São Paulo (IBSP); Museo Argentino de Ciencias Naturales Bernardino Rivadavia, Buenos Aires (MACN); Museo de Historia Natural de La Salle, Bogotá (MLS); Museo de Historia Natural de la Universidad de Cauca, Popayán (MHNUC); Museo de Historia Natural Noel Kempff Mercado, Santa Cruz (NK); Museo Nacional de Historia Natural del Paraguay, San Lorenzo (MNHNP); Museu de História Natural Capão da Imbuia, Curitiba (MHNCI); Museu Paraense Emílio Goeldi, Belém (MPEG); Museu de Zoologia da Universidade de São Paulo, São Paulo (MZUSP); Museu de Zoologia da Universidade Estadual de Santa Cruz, Ilhéus (MZUESC); Museu Nacional de História Natural, Rio de Janeiro (MNRJ); Museum für Naturkunde, Berlin (ZMB); Muséum National d'Histoire Naturelle, Paris (MNHN); Museum of Comparative Zoology, Cambridge (MCZ); Museum of Vertebratre Zoology, Berkeley (MVZ); National Museum of Natural History, Smithsonian Institution, Washington (USNM); Natural History Museum, London (BMNH); Naturhistorisches Museum Wien, Vienna (MNHNW); Naturhistoriska Riksmuseet, Stockholm (NHRM); Royal Ontario Museum, Toronto (ROM); Senckenberg Forschungsinstitut und Naturmuseum, Frankfurt (SMF); University of Kansas Biodiversity Institute, Lawrence (UK); University of Michigan Museum of Zoology, Ann Harbor (UIMNH); Uppsala University Museum of Evolution, Uppsala (UUZM); Zoologische Museum Hamburg, Hamburg (ZMH); Zoologische Staatssammlung München, Munich (ZSM).
