# Supplementary material for: Reducing Wallacean shortfalls for the coralsnakes of the Micrurus lemniscatus species complex: Present and future distributions under a changing climate
Source: PLoS One. 2018 Nov 14;13(11):e0205164. doi: 10.1371/journal.pone.0205164 (PMC6241113; doi:10.1371/journal.pone.0205164)
Supplement: S2 Appendix — Results of ENM and analyses for M. l. carvalhoi after controlling for spatial aggregation in occurrence records. (PDF) [file pone.0205164.s002.pdf]

S2 Appendix. Results of ENM for *M. l. carvalhoi*. Results of ENM and analyses for *M. l. carvalhoi* after controlling for spatial aggregation in occurrence records.

After applying the protocol described in the main text to reduce the spatial aggregation and autocorrelation in occurrence records for *M. l. carvalhoi*, construct ENM again, and redid the analyses related to range shifts and range representation into the protected areas (SRI). We found that the species will lost 47.05% of its current geographic distribution (against a loss of 32.9% in the previous analyses). Regarding the SRI, we found a slight difference: in previous analyses without controlling for spatial autocorrelation, the SRI was 0.14 for present and 0.13 for future. Now, it was 0.17 for present and 0.16 for future (i.e., SRI has increased, but it is still low). Overall, these results did not change the general tendencies in range shift and SRI observed for this species. Thus, in the main text, we present only the results from using all the occurrence dataset.
