# Supplementary material for: Reducing Wallacean shortfalls for the coralsnakes of the Micrurus lemniscatus species complex: Present and future distributions under a changing climate
Source: PLoS One. 2018 Nov 14;13(11):e0205164. doi: 10.1371/journal.pone.0205164 (PMC6241113; doi:10.1371/journal.pone.0205164)
Supplement: S1 Table — List of occurrence records with the collection IDs. Collection abbreviations correspond to the museums listed in the S1 Appendix above. (PDF) [file pone.0205164.s003.pdf]

S1 Table. List of occurrence records. List of occurrence records with the collection IDs. Collection abbreviations correspond to the museums listed in the S1 Appendix.

Species	Collection ID	LAT	LONG
M. diutius	INPA 11122	-3.320729°	-64.723583°
M. diutius	AMNH 137579	10.699402°	-61.293234°
M. diutius	MPEG 429	1.051882°	-51.933746°
M. diutius	ROM 43354	10.698202°	-61.296992°
M. diutius	AMNH 137576	10.699402°	-61.293234°
M. diutius	AMNH 75821	10.696790°	-61.291173°
M. diutius	MNHN 1962.0466	3.831487°	-51.835444°
M. diutius	MNHN 1997.3680	5.140278°	-52.931704°
M. diutius	MNHN 1989.3151	4.565928°	-52.407260°
M. diutius	MNHN 1994.8772	4.866666°	-53.066666°
M. diutius	MZUSP 9243	2.667000°	-61.250000°
M. diutius	USNM 252691	10.631110°	-61.275162°
M. diutius	CM 44325	5.442520°	-55.098965°
M. diutius	MNHN 0.7658	4.889220°	-52.314743°
M. diutius	MZUSP 10443	1.750632°	-62.276748°
M. diutius	UMMZ 123045	10.699402°	-61.293234°
M. diutius	AMNH 110157	10.692158°	-61.290956°
M. diutius	BMNH 1946.4.4.59	5.724689°	-55.668981°
M. diutius	FMNH 219619	10.659876°	-61.127284°
M. diutius	MZUSP 8824	4.477809°	-61.148143°
M. diutius	USNM 286963	10.770446°	-61.306084°
M. diutius	FMNH 34472	10.654826°	-61.385558°
M. diutius	MCZ 152689	5.201332°	-55.972105°
M. diutius	MZUSP 9258	2.667000°	-61.250000°
M. diutius	AMNH 101413	10.692158°	-61.290956°
M. diutius	AMNH 81428	10.593717°	-61.128593°
M. diutius	FMT 24	-3.060700°	-60.013000°
M. diutius	USNM 252692	10.631110°	-61.275162°
M. diutius	AMNH 114797	7.351681°	-62.535553°
M. diutius	FMNH 77898	10.094311°	-61.827046°
M. diutius	KU 117057	10.281118°	-64.401983°
M. diutius	MPEG 19693	0.983333°	-52.083333°
M. diutius	MZUSP 9723	2.833333°	-63.633333°
M. diutius	USNM 217257	6.170000°	-61.350000°
M. diutius	AMNH 119444	10.692158°	-61.290956°
M. diutius	AMNH 64487	10.713105°	-61.610123°
M. diutius	AMNH 64911	-3.060700°	-60.013000°
M. diutius	BMNH 1964.1933	10.672767°	-61.373756°
M. diutius	CMNH S 7925	10.247227°	-63.920293°
M. diutius	FMT 1445	-3.060700°	-60.013000°
M. diutius	FMT 3764	-3.060700°	-60.013000°
M. diutius	IBSP 40860	-3.060700°	-60.013000°
M. diutius	FMT 1698	-3.060700°	-60.013000°
M. diutius	FMT 3159	-3.060700°	-60.013000°
M. diutius	FMT 643	-2.034352°	-60.025875°
M. diutius	FMT 725	-3.060700°	-60.013000°
M. diutius	MPEG 21168	-1.759944°	-55.862470°
M. diutius	MZUSP 4792	-1.759944°	-55.862470°
M. diutius	MZUSP 5465	0.502478°	-63.393931°
M. diutius	AMNH 64912	-3.060700°	-60.013000°

M. diutius	INPA 17275	-3.060700°	-60.013000°
M. diutius	INPA 18768	-3.060700°	-60.013000°
M. diutius	MNRJ 14920	-1.491126°	-56.372931°
M. diutius	MPEG 17762	-2.034352°	-60.025875°
M. diutius	MPEG 22511	-2.153297°	-56.087232°
M. diutius	FMT 1005	-3.060700°	-60.013000°
M. diutius	FMT 1897	-3.060700°	-60.013000°
M. diutius	FMT 20	-1.759944°	-55.862470°
M. diutius	FMT 3150	-3.060700°	-60.013000°
M. diutius	FMT 347	-2.034352°	-60.025875°
M. diutius	MPEG 21169	-1.759944°	-55.862470°
M. diutius	FMT 2180	-3.060700°	-60.013000°
M. diutius	MPEG 17579	-2.034352°	-60.025875°
M. diutius	MPEG 19150	4.416218°	-61.140997°
M. diutius	USNM 314704	10.087153°	-61.729277°
M. diutius	MNHN 1994.8781	4.850000°	-53.066666°
M. diutius	FMT 164	-3.060700°	-60.013000°
M. diutius	FMT 2982	-3.060700°	-60.013000°
M. diutius	FMT 2037	-3.060700°	-60.013000°
M. diutius	FMT 2390	-3.060700°	-60.013000°
M. diutius	INPA 17641	-3.060700°	-60.013000°
M. diutius	FMT 134	-3.060700°	-60.013000°
M. diutius	FMT 3016	-3.060700°	-60.013000°
M. diutius	FMT 982	-3.060700°	-60.013000°
M. diutius	MPEG 21167	-1.759944°	-55.862470°
M. diutius	MPEG 22176	-1.759944°	-55.862470°
M. diutius	FMT 2498	-3.060700°	-60.013000°
M. diutius	USNM 252693	10.631110°	-61.275162°
M. diutius	FMT 1972	-3.060700°	-60.013000°
M. diutius	FMT 510	-3.060700°	-60.013000°
M. diutius	FMNH 35118	4.367869°	-57.939465°
M. diutius	MPEG 19692	0.983333°	-52.083333°
M. diutius	FMNH 251258	10.699402°	-61.293234°
M. diutius	FMNH 26659	6.468700°	-58.947589°
M. diutius	ROM 11702	2.572518°	-59.923464°
M. diutius	FMNH 223661	10.692158°	-61.290956°
M. diutius	FMNH 217240	10.699402°	-61.293234°
M. diutius	AMNH 137578	10.699402°	-61.293234°
M. diutius	FMNH 75950	10.659567°	-61.478912°
M. diutius	MPEG 22281	-1.759944°	-55.862470°
M. diutius	KU 167614	10.555749°	-63.122953°
M. diutius	MZUSP 10713	1.750632°	-62.276748°
M. diutius	MZUSP 9306	3.416667°	-61.666667°
M. diutius	AMNH 137575	10.699402°	-61.293234°
M. diutius	MPEG 19694	0.983333°	-52.083333°
M. diutius	AMNH 137577	10.699402°	-61.293234°
M. diutius	FMNH 75949	10.742829°	-61.608635°
M. diutius	FMT 731	-1.083333°	-57.033333°
M. diutius	IBSP 15077	-3.060700°	-60.013000°
M. diutius	IBSP 1728	-3.060700°	-60.013000°
M. diutius	MNHN 1900.0171	3.831487°	-51.835444°
M. diutius	AMNH 73091	10.622116°	-61.213023°
M. diutius	FMNH 77897	10.625762°	-61.058940°
M. diutius	FMT 104	-3.060700°	-60.013000°
M. diutius	FMT 2081	-3.060700°	-60.013000°
M. diutius	FMT 304	-2.034352°	-60.025875°

M. diutius	FMT 553	-2.034352°	-60.025875°
M. diutius	FMT 1820	-3.060700°	-60.013000°
M. diutius	FMT 2493	-3.060700°	-60.013000°
M. diutius	IBSP 52228	-2.034352°	-60.025875°
M. diutius	MPEG 17784	-2.034352°	-60.025875°
M. diutius	MPEG 23494	-1.759944°	-55.862470°
M. diutius	MZUSP 9224	3.300000°	-61.450000°
M. diutius	FMNH 219607	10.692158°	-61.290956°
M. diutius	FMT 2269	2.671667°	-61.450610°
M. diutius	MPEG 23769	-3.141981°	-58.442635°
M. diutius	ZSM 194/1909	10.608141°	-61.190273°
M. diutius	FMT 1953	-3.060700°	-60.013000°
M. diutius	USNM 166720	10.54167°	-61.13492°
M. diutius	FMT 72	-3.060700°	-60.013000°
M. diutius	FMT 973	-3.054651°	-59.942143°
M. diutius	ROM 22834	7.366667°	-60.483333°
M. diutius	FMNH 75957	10.593717°	-61.128593°
M. diutius	MPEG 20787	-1.759944°	-55.862470°
M. l. carvalhoi	FMNH 37739	-22.746792°	-48.582653°
M. l. carvalhoi	IBSP 49285	-21.176657°	-47.820762°
M. l. carvalhoi	IBSP 65555	-10.168891°	-48.331717°
M. l. carvalhoi	IBSP 937	-21.995955°	-47.426824°
M. l. carvalhoi	IBSP 9913	-22.352118°	-48.775239°
M. l. carvalhoi	IBSP 42655	-21.238186°	-48.809693°
M. l. carvalhoi	IBSP 9980	-21.995955°	-47.426824°
M. l. carvalhoi	MZUSP 4901	-21.933333°	-47.366667°
M. l. carvalhoi	IBSP 34140	-14.197054°	-41.669309°
M. l. carvalhoi	IBSP 3041	-23.548943°	-46.638818°
M. l. carvalhoi	IBSP 409	-22.431990°	-46.958156°
M. l. carvalhoi	IBSP 51258	-22.660969°	-50.399546°
M. l. carvalhoi	IBSP 7197	-23.045867°	-49.169137°
M. l. carvalhoi	IBSP 30158	-22.413399°	-47.569574°
M. l. carvalhoi	IBSP 3050	-23.548943°	-46.638818°
M. l. carvalhoi	IBSP 30691	-21.764210°	-43.349570°
M. l. carvalhoi	IBSP 4628	-23.548943°	-46.638818°
M. l. carvalhoi	IBSP 51151	-22.315443°	-49.061465°
M. l. carvalhoi	IBSP 59640	-22.500000°	-53.016667°
M. l. carvalhoi	IBSP 71005	-21.995955°	-47.426824°
M. l. carvalhoi	IBSP 16235	-25.591735°	-49.411538°
M. l. carvalhoi	IBSP 16827	-22.315443°	-49.061465°
M. l. carvalhoi	IBSP 41229	-21.176657°	-47.820762°
M. l. carvalhoi	IBSP 41789	-21.764210°	-43.349570°
M. l. carvalhoi	IBSP 42529	-21.764210°	-43.349570°
M. l. carvalhoi	IBSP 44338	-22.595994°	-48.811240°
M. l. carvalhoi	IBSP 46388	-22.907686°	-49.617565°
M. l. carvalhoi	IBSP 50144	-22.015354°	-47.891112°
M. l. carvalhoi	IBSP 5267	-25.586442°	-49.632028°
M. l. carvalhoi	IBSP 61489	-23.342359°	-49.374748°
M. l. carvalhoi	IBSP 7651	-23.543333°	-46.410833°
M. l. carvalhoi	IBSP 7748	-21.994863°	-48.391535°
M. l. carvalhoi	IBSP 1547	-21.467146°	-48.389024°
M. l. carvalhoi	IBSP 44451	-22.189237°	-47.397999°
M. l. carvalhoi	IBSP 51260	-22.499393°	-48.552294°
M. l. carvalhoi	IBSP 7754	-21.916667°	-49.250000°
M. l. carvalhoi	ZUFMS REP 2017	-20.443505°	-54.647759°
M. l. carvalhoi	CZGB 2758	-23.506229°	-47.455910°

M. l. carvalhoi	IBSP 1044	-21.346590°	-43.050452°
M. l. carvalhoi	IBSP 16083	-22.064114°	-48.174710°
M. l. carvalhoi	IBSP 1613	-20.891631°	-47.585646°
M. l. carvalhoi	IBSP 29827	-21.688138°	-48.081527°
M. l. carvalhoi	IBSP 43226	-21.176657°	-47.820762°
M. l. carvalhoi	IBSP 43601	-22.015354°	-47.891112°
M. l. carvalhoi	IBSP 53009	-22.739486°	-49.731852°
M. l. carvalhoi	IBSP 56234	-22.595994°	-48.811240°
M. l. carvalhoi	IBSP 9672	-21.478101°	-47.550675°
M. l. carvalhoi	IBSP 9946	-26.230389°	-51.086632°
M. l. carvalhoi	IBSP 9967	-20.720394°	-47.887598°
M. l. carvalhoi	MZUESC 3071	-17.872155°	-39.385588°
M. l. carvalhoi	CCG 006	-27.362137°	-55.900875°
M. l. carvalhoi	IBSP 16672	-21.176657°	-47.820762°
M. l. carvalhoi	IBSP 23676	-21.794613°	-48.176593°
M. l. carvalhoi	IBSP 28676	-22.357732°	-47.384946°
M. l. carvalhoi	IBSP 33703	-23.194308°	-49.384419°
M. l. carvalhoi	IBSP 40211	-21.764210°	-43.349570°
M. l. carvalhoi	IBSP 43830	-22.176753°	-55.939853°
M. l. carvalhoi	IBSP 44427	-21.420911°	-50.078045°
M. l. carvalhoi	IBSP 51312	-21.995955°	-47.426824°
M. l. carvalhoi	IBSP 70970	-21.995955°	-47.426824°
M. l. carvalhoi	IBSP 9889	-20.100000°	-47.800000°
M. l. carvalhoi	CEPB 892	-18.948989°	-51.908812°
M. l. carvalhoi	IBSP 12526	-22.766667°	-47.950000°
M. l. carvalhoi	IBSP 1300	-22.431990°	-46.958156°
M. l. carvalhoi	IBSP 18290	-20.949564°	-48.478863°
M. l. carvalhoi	IBSP 18724	-21.023927°	-47.372695°
M. l. carvalhoi	IBSP 37264	-20.177679°	-48.033444°
M. l. carvalhoi	IBSP 40203	-21.764210°	-43.349570°
M. l. carvalhoi	IBSP 41791	-21.176657°	-47.820762°
M. l. carvalhoi	IBSP 42369	-21.176657°	-47.820762°
M. l. carvalhoi	IBSP 46048	-20.559217°	-48.568862°
M. l. carvalhoi	IBSP 50883	-22.499393°	-48.552294°
M. l. carvalhoi	IBSP 55748	-23.104485°	-48.939697°
M. l. carvalhoi	IBSP 60322	-23.045867°	-49.169137°
M. l. carvalhoi	IBSP 70	-23.548943°	-46.638818°
M. l. carvalhoi	IBSP 7018	-20.949564°	-48.478863°
M. l. carvalhoi	IBSP 7447	-20.870800°	-51.486932°
M. l. carvalhoi	IBSP 9928	-21.250000°	-47.400000°
M. l. carvalhoi	CHINM 3382	-27.362137°	-55.900875°
M. l. carvalhoi	IBSP 23678	-18.749166°	-44.446755°
M. l. carvalhoi	IBSP 27819	-23.799053°	-48.587432°
M. l. carvalhoi	IBSP 47354	-18.236555°	-47.842628°
M. l. carvalhoi	IBSP 49507	-20.891631°	-47.585646°
M. l. carvalhoi	IBSP 5266	-25.586442°	-49.632028°
M. l. carvalhoi	IBSP 67833	-23.194308°	-49.384419°
M. l. carvalhoi	IBSP 71008	-21.903960°	-47.619870°
M. l. carvalhoi	MCZ 17852	-19.596848°	-42.291309°
M. l. carvalhoi	MZUSP 10812	-27.951714°	-58.804085°
M. l. carvalhoi	USNM 76341	-21.138258°	-48.973762°
M. l. carvalhoi	USNM 76343	-21.138258°	-48.973762°
M. l. carvalhoi	IBSP 19115	-21.023927°	-47.372695°
M. l. carvalhoi	IBSP 28475	-21.255390°	-48.322418°
M. l. carvalhoi	IBSP 30665	-22.660969°	-50.399546°
M. l. carvalhoi	IBSP 30730	-20.298078°	-45.545732°

M. l. carvalhoi	IBSP 41797	-21.176657°	-47.820762°
M. l. carvalhoi	IBSP 46675	-22.890396°	-48.455309°
M. l. carvalhoi	IBSP 4759	-23.548943°	-46.638818°
M. l. carvalhoi	IBSP 50538	-23.002787°	-49.324606°
M. l. carvalhoi	IBSP 55465	-22.469143°	-48.989531°
M. l. carvalhoi	IBSP 5801	-21.467146°	-48.389024°
M. l. carvalhoi	IBSP 10382	-15.767847°	-47.919327°
M. l. carvalhoi	IBSP 18619	-20.720358°	-46.610090°
M. l. carvalhoi	IBSP 29734	-21.995955°	-47.426824°
M. l. carvalhoi	IBSP 3055	-23.548943°	-46.638818°
M. l. carvalhoi	IBSP 30621	-21.008009°	-42.718376°
M. l. carvalhoi	IBSP 43227	-21.995955°	-47.426824°
M. l. carvalhoi	IBSP 44240	-22.738788°	-47.331913°
M. l. carvalhoi	IBSP 53069	-21.176657°	-47.820762°
M. l. carvalhoi	IBSP 7814	-20.870800°	-51.486932°
M. l. carvalhoi	MZUESC 856	-17.872155°	-39.385588°
M. l. carvalhoi	MZUSP 10135	-21.127032°	-55.832099°
M. l. carvalhoi	USNM 39074	-19.596848°	-42.291309°
M. l. carvalhoi	CHINM 2403	-28.112778°	-55.630278°
M. l. carvalhoi	IBSP 29146	-21.764210°	-43.349570°
M. l. carvalhoi	IBSP 40250	-21.764210°	-43.349570°
M. l. carvalhoi	IBSP 41871	-20.949564°	-48.478863°
M. l. carvalhoi	IBSP 42277	-21.238186°	-48.809693°
M. l. carvalhoi	IBSP 42588	-19.491374°	-44.390978°
M. l. carvalhoi	IBSP 7526	-21.021353°	-48.050378°
M. l. carvalhoi	ZUFMS REP 0131	-20.443505°	-54.647759°
M. l. carvalhoi	IBSP 1593	-21.143404°	-48.007032°
M. l. carvalhoi	IBSP 21138	-21.346590°	-43.050452°
M. l. carvalhoi	IBSP 50968	-21.470827°	-47.000569°
M. l. carvalhoi	IBSP 51152	-22.761653°	-47.154074°
M. l. carvalhoi	IBSP 5724	-20.650000°	-47.550000°
M. l. carvalhoi	IBSP 8333	-20.870800°	-51.486932°
M. l. carvalhoi	MZUSP 20432	-7.161999°	-35.051831°
M. l. carvalhoi	USNM 76340	-21.138258°	-48.973762°
M. l. carvalhoi	IBSP 31050	-21.764210°	-43.349570°
M. l. carvalhoi	IBSP 33067	-23.716667°	-48.450000°
M. l. carvalhoi	IBSP 33324	-21.764210°	-43.349570°
M. l. carvalhoi	IBSP 50164	-17.282955°	-42.735391°
M. l. carvalhoi	IBSP 5976	-25.397038°	-51.236109°
M. l. carvalhoi	CHINM 1473	-27.362137°	-55.900875°
M. l. carvalhoi	IBSP 32276	-21.764210°	-43.349570°
M. l. carvalhoi	IBSP 40628	-20.917319°	-46.991410°
M. l. carvalhoi	IBSP 47504	-19.530631°	-45.952705°
M. l. carvalhoi	IBSP 65034	-9.751412°	-48.357532°
M. l. carvalhoi	IBSP 67825	-23.194308°	-49.384419°
M. l. carvalhoi	IBSP 7630	-21.195481°	-46.962782°
M. l. carvalhoi	IBSP 7841	-22.898056°	-48.491389°
M. l. carvalhoi	IBSP 900	-22.366667°	-47.350000°
M. l. carvalhoi	IBSP 27452	-23.104485°	-48.939697°
M. l. carvalhoi	IBSP 28688	-22.357732°	-47.384946°
M. l. carvalhoi	IBSP 30903	-23.098212°	-48.258828°
M. l. carvalhoi	IBSP 32855	-21.764210°	-43.349570°
M. l. carvalhoi	IBSP 40308	-21.764210°	-43.349570°
M. l. carvalhoi	IBSP 40352	-21.764210°	-43.349570°
M. l. carvalhoi	IBSP 45516	-22.660969°	-50.399546°
M. l. carvalhoi	MNRJ 1317	-19.629075°	-43.889747°

M. l. carvalhoi	IBSP 33312	-21.764210°	-43.349570°
M. l. carvalhoi	IBSP 43221	-21.185700°	-42.385900°
M. l. carvalhoi	IBSP 45884	-21.708146°	-45.269536°
M. l. carvalhoi	MNHNP 5145	-25.335116°	-55.717372°
M. l. carvalhoi	MNRJ 17492	-22.786746°	-43.313176°
M. l. carvalhoi	IBSP 32383	-21.764210°	-43.349570°
M. l. carvalhoi	IBSP 42578	-21.764210°	-43.349570°
M. l. carvalhoi	IBSP 50712	-20.720394°	-47.887598°
M. l. carvalhoi	IBSP 33464	-21.764210°	-43.349570°
M. l. carvalhoi	IBSP 8978	-20.541597°	-47.421637°
M. l. carvalhoi	CEPB 7440	-16.751810°	-48.518817°
M. l. carvalhoi	IBSP 31052	-20.754590°	-42.882524°
M. l. carvalhoi	IBSP 32957	-21.764210°	-43.349570°
M. l. carvalhoi	IBSP 51012	-20.917319°	-46.991410°
M. l. carvalhoi	AMNH 96999	-10.655774°	-50.604949°
M. l. carvalhoi	IBSP 30785	-21.764210°	-43.349570°
M. l. carvalhoi	IBSP 31007	-21.764210°	-43.349570°
M. l. carvalhoi	CEPB 13300 (n° campo)	-12.232824°	-48.392745°
M. l. carvalhoi	IBSP 30789	-21.764210°	-43.349570°
M. l. carvalhoi	IBSP 57227	-21.794613°	-48.176593°
M. l. carvalhoi	IBSP 65556	-10.168891°	-48.331717°
M. l. carvalhoi	MZUSP 15534	-12.232824°	-48.392745°
M. l. carvalhoi	IBSP 32962	-21.764210°	-43.349570°
M. l. carvalhoi	IBSP 34448	-21.764210°	-43.349570°
M. l. carvalhoi	IBSP 41166	-21.764210°	-43.349570°
M. l. carvalhoi	IBSP 53277	-23.135726°	-49.054561°
M. l. carvalhoi	CHINM 3043	-28.112778°	-55.630278°
M. l. carvalhoi	IBSP 43602	-21.764210°	-43.349570°
M. l. carvalhoi	MNHCI 4541	-23.259861°	-55.540004°
M. l. carvalhoi	MZUESC 855	-17.872155°	-39.385588°
M. l. carvalhoi	IBSP 3046	-23.548943°	-46.638818°
M. l. carvalhoi	IBSP 41159	-22.761653°	-47.154074°
M. l. carvalhoi	IBSP 26966	-20.917319°	-46.991410°
M. l. carvalhoi	MNRJ 1318	-19.629075°	-43.889747°
M. l. carvalhoi	IBSP 49290	-20.038247°	-47.747914°
M. l. carvalhoi	CZGB 8319	-17.872155°	-39.385588°
M. l. carvalhoi	MZUSP 14517	-9.750000°	-48.399999°
M. l. carvalhoi	IBSP 49389	-21.176657°	-47.820762°
M. l. carvalhoi	IBSP 66021	-10.707530°	-48.414268°
M. l. carvalhoi	IBSP 67556	-10.707530°	-48.414268°
M. l. carvalhoi	MZUSP 14518	-9.750000°	-48.399999°
M. l. carvalhoi	MZUSP 71	-20.559217°	-48.568862°
M. l. carvalhoi	IBSP 66020	-10.707530°	-48.414268°
M. l. carvalhoi	IBSP 65554	-10.168891°	-48.331717°
M. l. carvalhoi	IBSP 67067	-10.168891°	-48.331717°
M. l. carvalhoi	IBSP 16611	-20.998147°	-48.215452°
M. l. carvalhoi	IBSP 32363	-21.764210°	-43.349570°
M. l. carvalhoi	IBSP 33586	-21.764210°	-43.349570°
M. l. carvalhoi	IBSP 46467	-21.532306°	-46.649867°
M. l. carvalhoi	IBSP 65035	-10.707530°	-48.414268°
M. l. carvalhoi	IBSP 67555	-10.707530°	-48.414268°
M. l. carvalhoi	MNRJ 957	-10.655774°	-50.604949°
M. l. carvalhoi	IBSP 52202	-21.176657°	-47.820762°
M. l. carvalhoi	IBSP 5770	-22.602875°	-49.000077°
M. l. carvalhoi	IBSP 53302	-21.176657°	-47.820762°
M. l. carvalhoi	IBSP 28272	-21.764210°	-43.349570°

M. l. carvalhoi	IBSP 51611	-21.903960°	-47.619870°
M. l. carvalhoi	IBSP 34415	-21.764210°	-43.349570°
M. l. carvalhoi	IBSP 32123	-21.688138°	-48.081527°
M. l. carvalhoi	IBSP 45495	-22.761653°	-47.154074°
M. l. carvalhoi	CEPB 3140	-18.948989°	-51.908812°
M. l. carvalhoi	IBSP 30589	-21.688138°	-48.081527°
M. l. carvalhoi	IBSP 33508	-23.104485°	-48.939697°
M. l. carvalhoi	IBSP 41395	-21.176657°	-47.820762°
M. l. carvalhoi	IBSP 43835	-21.764210°	-43.349570°
M. l. carvalhoi	IBSP 31051	-21.764210°	-43.349570°
M. l. carvalhoi	IBSP 53072	-21.995955°	-47.426824°
M. l. carvalhoi	IBSP 6064	-23.117116°	-46.550216°
M. l. carvalhoi	IBSP 40317	-21.764210°	-43.349570°
M. l. carvalhoi	IBSP 49292	-20.038247°	-47.747914°
M. l. carvalhoi	IBSP 53303	-22.189237°	-47.397999°
M. l. carvalhoi	IBSP 41074	-21.764210°	-43.349570°
M. l. carvalhoi	IBSP 43613	-21.764210°	-43.349570°
M. l. carvalhoi	IBSP 31382	-21.764210°	-43.349570°
M. l. carvalhoi	IBSP 52241	-21.176657°	-47.820762°
M. l. carvalhoi	IBSP 40724	-21.764210°	-43.349570°
M. l. carvalhoi	MZUSP 2944	-9.933333°	-36.083333°
M. l. carvalhoi	IBSP 44642	-22.660969°	-50.399546°
M. l. carvalhoi	IBSP 42532	-21.764210°	-43.349570°
M. l. carvalhoi	IBSP 51891	-21.731180°	-47.495930°
M. l. carvalhoi	IBSP 56467	-16.680171°	-49.254888°
M. l. carvalhoi	IBSP 59666	-16.678994°	-50.456016°
M. l. carvalhoi	IBSP 30643	-21.848352°	-43.807533°
M. l. carvalhoi	IBSP 42457	-20.283447°	-50.246597°
M. l. carvalhoi	IBSP 46975	-21.771280°	-47.091037°
M. l. carvalhoi	IBSP 51599	-21.778297°	-48.559281°
M. l. carvalhoi	FMNH 42194	-8.057581°	-34.888922°
M. l. carvalhoi	IBSP 46339	-20.727578°	-48.053884°
M. l. carvalhoi	IBSP 50532	-21.794613°	-48.176593°
M. l. carvalhoi	IBSP 53074	-23.041491°	-49.721764°
M. l. carvalhoi	IBSP 40146	-22.412422°	-49.136512°
M. l. carvalhoi	IBSP 16703	-21.848352°	-43.807533°
M. l. carvalhoi	IBSP 30947	-23.548943°	-46.638818°
M. l. carvalhoi	IBSP 32137	-19.829462°	-42.316652°
M. l. carvalhoi	IBSP 40740	-21.764210°	-43.349570°
M. l. carvalhoi	IBSP 43807	-23.285429°	-47.674020°
M. l. carvalhoi	IBSP 31726	-23.356834°	-47.857391°
M. l. carvalhoi	IBSP 43849	-22.761653°	-47.154074°
M. l. carvalhoi	IBSP 22397	-21.536513°	-49.859570°
M. l. carvalhoi	IBSP 41795	-21.176657°	-47.820762°
M. l. carvalhoi	IBSP 50696	-21.470827°	-47.000569°
M. l. carvalhoi	IBSP 71006	-21.995955°	-47.426824°
M. l. carvalhoi	FMNH 42193	-8.057581°	-34.888922°
M. l. carvalhoi	IBSP 30641	-21.848352°	-43.807533°
M. l. carvalhoi	IBSP 30687	-21.764210°	-43.349570°
M. l. carvalhoi	IBSP 37440	-20.559217°	-48.568862°
M. l. carvalhoi	IBSP 40113	-21.549518°	-47.706674°
M. l. carvalhoi	IBSP 45689	-21.021353°	-48.050378°
M. l. carvalhoi	IBSP 53002	-21.596130°	-48.813447°
M. l. carvalhoi	IBSP 55254	-19.097156°	-46.673720°
M. l. carvalhoi	MZUSP 8641	-18.489136°	-54.753813°
M. l. carvalhoi	IBSP 30049	-21.764210°	-43.349570°

M. l. carvalhoi	IBSP 30729	-21.764210°	-43.349570°
M. l. carvalhoi	IBSP 33118	-21.764210°	-43.349570°
M. l. carvalhoi	IBSP 41358	-20.720394°	-47.887598°
M. l. carvalhoi	IBSP 53073	-15.767847°	-47.919327°
M. l. carvalhoi	IBSP 57136	-21.660988°	-45.925027°
M. l. carvalhoi	IBSP 43692	-18.421614°	-49.215522°
M. l. carvalhoi	MZUSP 72	-20.559217°	-48.568862°
M. l. carvalhoi	IBSP 29404	-21.008009°	-42.718376°
M. l. carvalhoi	IBSP 965	-17.714448°	-39.511249°
M. l. carvalhoi	IBSP 31132	-21.764210°	-43.349570°
M. l. carvalhoi	IBSP 54711	-21.729073°	-43.067314°
M. l. carvalhoi	MZUSP 2972	-20.720358°	-46.610090°
M. l. carvalhoi	IBSP 30639	-21.848352°	-43.807533°
M. l. carvalhoi	IBSP 33545	-21.764210°	-43.349570°
M. l. carvalhoi	IBSP 33592	-21.764210°	-43.349570°
M. l. carvalhoi	IBSP 30727	-21.764210°	-43.349570°
M. l. carvalhoi	IBSP 51952	-21.176657°	-47.820762°
M. l. carvalhoi	NHMW 18640	-15.598917°	-56.094894°
M. l. carvalhoi	IBSP 22865	-20.720358°	-46.610090°
M. l. carvalhoi	USNM 100718	-20.377999°	-43.416683°
M. l. carvalhoi	AMNH 119215	-9.933333°	-36.083333°
M. l. carvalhoi	USNM 76342	-21.138258°	-48.973762°
M. l. carvalhoi	IBSP 34485	-21.764210°	-43.349570°
M. l. carvalhoi	IBSP 62436	-21.660988°	-45.925027°
M. l. carvalhoi	IBSP 42523	-21.764210°	-43.349570°
M. l. carvalhoi	IBSP 42544	-21.764210°	-43.349570°
M. l. carvalhoi	IBSP 65036	-10.707530°	-48.414268°
M. l. helleri	BMNH 1913.7.28.20	-9.414813°	-73.428485°
M. l. helleri	BMNH 1874.8.4.44	-6.032993°	-76.971313°
M. l. helleri	MLS 1542	4.269624°	-73.567932°
M. l. helleri	FMNH 45624	-8.363156°	-74.567716°
M. l. helleri	IAvH-R 1928	-1.236390°	-69.921890°
M. l. helleri	AMNH 110449	-12.473045°	-64.212606°
M. l. helleri	AMNH 55922	-5.018685°	-73.578398°
M. l. helleri	USNM 566627	-4.014700°	-77.778600°
M. l. helleri	AMNH 55771	-10.983333°	-75.416667°
M. l. helleri	BMNH 1874.8.4.48	-6.032993°	-76.971313°
M. l. helleri	BMNH 1874.8.4.55	-6.032993°	-76.971313°
M. l. helleri	NRM 6308	-1.821678°	-77.706035°
M. l. helleri	MHNUC-He-Se-R 319	4.150000°	-73.633333°
M. l. helleri	MHNUC-He-Se-R 484	1.200000°	-70.166667°
M. l. helleri	INPA 15758	-0.119354°	-67.082435°
M. l. helleri	UMMZ 57696	-10.416197°	-65.395549°
M. l. helleri	ZMH-R 2418	-14.283599°	-66.266486°
M. l. helleri	MLS 1535	4.150000°	-73.633333°
M. l. helleri	MLS 1537	4.150000°	-73.633333°
M. l. helleri	USNM 193723	-9.316667°	-76.033333°
M. l. helleri	MPEG 20373	-10.790926°	-65.332216°
M. l. helleri	USNM 193720	-9.050000°	-75.583333°
M. l. helleri	MLS 1531	1.750000°	-75.583333°
M. l. helleri	BMNH 1874.8.4.47	-6.032993°	-76.971313°
M. l. helleri	CEPB 1722	-8.781712°	-63.702289°
M. l. helleri	CEPB 1869	-8.761825°	-63.901960°
M. l. helleri	FMNH 152316	-13.041418°	-64.668065°
M. l. helleri	AMNH 35816	-2.306485°	-78.118900°
M. l. helleri	ICN 11526	5.881877°	-71.893372°

M. l. helleri	ICN 7118	4.150000°	-73.633333°
M. l. helleri	KU 126059	0.074611°	-76.757917°
M. l. helleri	KU 222368	-3.233333°	-72.916667°
M. l. helleri	MLS 1532	1.360000°	-75.244600°
M. l. helleri	MLS 1539	4.150000°	-73.633333°
M. l. helleri	USNM 193722	-9.285492°	-76.010848°
M. l. helleri	SMF 20770	-10.461966°	-73.949737°
M. l. helleri	IBSP 52702	-8.761825°	-63.901960°
M. l. helleri	MLS 1536	4.150000°	-73.633333°
M. l. helleri	MLS 1540	4.150000°	-73.633333°
M. l. helleri	ICN 6880	4.508051°	-73.360443°
M. l. helleri	BMNH 1880.12.8.131	-1.589437°	-77.746597°
M. l. helleri	ICN 8168	1.012487°	-71.290855°
M. l. helleri	ICN 8262	4.150000°	-73.633333°
M. l. helleri	MZUSP 6108	4.050600°	-73.741290°
M. l. helleri	USNM 283971	-2.460000°	-78.170000°
M. l. helleri	IAvH-R 0189	3.350000°	-73.883333°
M. l. helleri	MLS 1541	4.269624°	-73.567932°
M. l. helleri	USNM 232439	-1.916667°	-77.23333°
M. l. helleri	MLS 1533	4.150000°	-73.633333°
M. l. helleri	ICN 34	-0.898286°	-70.454381°
M. l. helleri	USNM 283970	-3.283333°	-78.616667°
M. l. helleri	ICN 10555	-4.205429°	-69.932808°
M. l. helleri	ICN 25	0.070936°	-71.137368°
M. l. helleri	FMNH 5577	-10.068040°	-75.551666°
M. l. helleri	MZUSP 8357	-9.745309°	-63.283399°
M. l. helleri	IAvH-R 0794	-4.205429°	-69.932808°
M. l. helleri	IBSP 42704	-4.169749°	-69.946930°
M. l. helleri	SMF 20769	-10.461966°	-73.949737°
M. l. helleri	MLS 2967	4.269624°	-73.567932°
M. l. helleri	MZUSP 17351	-4.169749°	-69.946930°
M. l. helleri	AMNH 22483	-10.537146°	-65.583457°
M. l. helleri	MZUSP 18622	-9.197474°	-63.165502°
M. l. helleri	AMNH 55738	-10.983333°	-75.416667°
M. l. helleri	MLS 1887	4.269624°	-73.567932°
M. l. helleri	IBSP 28926	-4.160286°	-69.434312°
M. l. helleri	ICN 10906	3.987778°	-73.759722°
M. l. helleri	MPEG 16833	-10.711721°	-62.254971°
M. l. helleri	MCZ 173826	-1.053408°	-77.602188°
M. l. helleri	MVZ 199462	-12.600000°	-69.072890°
M. l. helleri	MLS 2196	6.783300°	-72.083300°
M. l. helleri	USNM 287946	-1.233333°	-77.416667°
M. l. helleri	AMNH 53462	-4.436324°	-77.660767°
M. l. helleri	MLS 1525	-4.205429°	-69.932808°
M. l. helleri	MLS 1530	1.750000°	-75.583333°
M. l. helleri	USNM 232440	-1.483003°	-78.002427°
M. l. helleri	USNM 566626	-4.014700°	-77.778600°
M. l. helleri	AMNH 126458	-9.285492°	-76.010848°
M. l. helleri	MLS 2526	4.150000°	-73.633333°
M. l. helleri	AMNH 36029	-1.589454°	-77.746605°
M. l. helleri	MZUSP 17352	-4.169749°	-69.946930°
M. l. helleri	USNM 316648	-4.933333°	-76.466667°
M. l. helleri	USNM 232438	-1.916667°	-77.23333°
M. l. helleri	MVZ 163326	-4.296280°	-78.244500°
M. l. helleri	AMNH 110450	-12.473045°	-64.212606°
M. l. helleri	ICN 31	-2.895630°	-69.750080°

M. l. lemniscatus	MPEG AL13	-2.153297°	-56.087232°
M. l. lemniscatus	MPEG 18657	-1.805694°	-50.714616°
M. l. lemniscatus	IBSP 48233	-3.768933°	-49.673651°
M. l. lemniscatus	MPEG 20083	-1.805694°	-50.714616°
M. l. lemniscatus	MPEG 2618	-1.455020°	-48.502368°
M. l. lemniscatus	ZSM 778/1920	-1.998946°	-54.082891°
M. l. lemniscatus	MPEG 18687	-1.518908°	-48.617001°
M. l. lemniscatus	AMNH 52610	-6.233508°	-74.037539°
M. l. lemniscatus	IBSP 47040	-3.768933°	-49.673651°
M. l. lemniscatus	MPEG 15280	-1.816667°	-46.283333°
M. l. lemniscatus	UMMZ 77508	6.010656°	-58.314796°
M. l. lemniscatus	IBSP 48954	-3.768933°	-49.673651°
M. l. lemniscatus	NHMW 13384:1	-4.388354°	-59.594486°
M. l. lemniscatus	AMNH 14148	6.383886°	-58.695553°
M. l. lemniscatus	BMNH 1923.11.9.137	-1.000000°	-49.500000°
M. l. lemniscatus	MPEG 388	-1.455020°	-48.502368°
M. l. lemniscatus	IBSP 47177	-3.768933°	-49.673651°
M. l. lemniscatus	IBSP 47179	-3.768933°	-49.673651°
M. l. lemniscatus	MPEG 18498	-1.518908°	-48.617001°
M. l. lemniscatus	MPEG 23798	-5.370657°	-49.118699°
M. l. lemniscatus	MPEG 266	-1.366384°	-48.372231°
M. l. lemniscatus	UMMZ 56891	-9.692407°	-65.436526°
M. l. lemniscatus	AMNH 14142	6.383886°	-58.695553°
M. l. lemniscatus	AMNH 14147	6.383886°	-58.695553°
M. l. lemniscatus	IBSP 48590	-3.768933°	-49.673651°
M. l. lemniscatus	MPEG 13001	-1.065952°	-46.789510°
M. l. lemniscatus	MPEG 13269	-1.297338°	-47.922144°
M. l. lemniscatus	MPEG 23544	-4.884496°	-65.311163°
M. l. lemniscatus	MPEG 23800	-5.369967°	-49.116928°
M. l. lemniscatus	MPEG 8455	-1.065952°	-46.789510°
M. l. lemniscatus	MZUSP 10444	1.750632°	-62.276748°
M. l. lemniscatus	ZSM 270/1989	-1.000000°	-49.500000°
M. l. lemniscatus	IBSP 47176	-3.768933°	-49.673651°
M. l. lemniscatus	IBSP 47785	-3.768933°	-49.673651°
M. l. lemniscatus	MPEG 19054	-2.440559°	-54.698575°
M. l. lemniscatus	MPEG 24063	-5.370657°	-49.118699°
M. l. lemniscatus	AMNH 14143	6.383886°	-58.695553°
M. l. lemniscatus	IBSP 46763	-3.768933°	-49.673651°
M. l. lemniscatus	IBSP 47180	-3.768933°	-49.673651°
M. l. lemniscatus	MPEG 16408	-1.455020°	-48.502368°
M. l. lemniscatus	MPEG 19772	-1.759944°	-55.862470°
M. l. lemniscatus	MPEG 22401	-3.194597°	-52.209315°
M. l. lemniscatus	MPEG 3044	-1.065952°	-46.789510°
M. l. lemniscatus	MPEG 5572	-1.297338°	-47.922144°
M. l. lemniscatus	ZMB 26425	5.866667°	-55.166667°
M. l. lemniscatus	AMNH 110158	6.383886°	-58.695553°
M. l. lemniscatus	AMNH 14146	6.383886°	-58.695553°
M. l. lemniscatus	AMNH 14251	6.383333°	-58.683333°
M. l. lemniscatus	MPEG 17144	-5.370657°	-49.118699°
M. l. lemniscatus	MPEG 20458	-1.805694°	-50.714616°
M. l. lemniscatus	USNM 13823	5.823201°	-55.167880°
M. l. lemniscatus	MPEG 16304	-1.192609°	-46.138680°
M. l. lemniscatus	MPEG 18686	-1.932977°	-47.049594°
M. l. lemniscatus	MPEG 20001	-1.805694°	-50.714616°
M. l. lemniscatus	MPEG 23146	-1.942295°	-50.809065°
M. l. lemniscatus	MPEG 23147	-1.942295°	-50.809065°

M. l. lemniscatus	MPEG 23358	-3.194597°	-52.209315°
M. l. lemniscatus	MZUSP 9411	-3.400000°	-51.883333°
M. l. lemniscatus	MPEG 16164	-3.461651°	-44.784000°
M. l. lemniscatus	MPEG 18698	-1.455020°	-48.502368°
M. l. lemniscatus	MPEG 18873	-1.805694°	-50.714616°
M. l. lemniscatus	MPEG 5557	-1.192609°	-46.138680°
M. l. lemniscatus	MPEG 8886	-1.816667°	-46.283333°
M. l. lemniscatus	IBSP 46766	-3.768933°	-49.673651°
M. l. lemniscatus	MPEG 13905	-1.816667°	-46.283333°
M. l. lemniscatus	MPEG 1516	-1.116667°	-48.400000°
M. l. lemniscatus	MPEG 16024	-1.192609°	-46.138680°
M. l. lemniscatus	MPEG 3043	-1.065952°	-46.789510°
M. l. lemniscatus	MPEG 5026	-1.065952°	-46.789510°
M. l. lemniscatus	MPEG 8890	-1.192609°	-46.138680°
M. l. lemniscatus	FMT 511	-2.034352°	-60.025875°
M. l. lemniscatus	IBSP 40213	-6.641410°	-51.978977°
M. l. lemniscatus	MPEG 1371	-1.024141°	-46.653811°
M. l. lemniscatus	MPEG 1515	-1.192609°	-46.138680°
M. l. lemniscatus	MPEG 15281	-1.816667°	-46.283333°
M. l. lemniscatus	MPEG 219	-1.366384°	-48.372231°
M. l. lemniscatus	MPEG 23237	-6.070741°	-49.904335°
M. l. lemniscatus	MPEG 3220	-1.024141°	-46.653811°
M. l. lemniscatus	MPEG 5533	-1.192609°	-46.138680°
M. l. lemniscatus	MPEG 8850	-1.024141°	-46.653811°
M. l. lemniscatus	UMMZ 80426	6.804496°	-58.154883°
M. l. lemniscatus	AMNH 130533	5.700000°	-55.233333°
M. l. lemniscatus	IBSP 41456	2.224694°	-55.947346°
M. l. lemniscatus	MPEG 23387	-2.153297°	-56.087232°
M. l. lemniscatus	MPEG 3765	-1.192609°	-46.138680°
M. l. lemniscatus	MPEG 5382	-1.024141°	-46.653811°
M. l. lemniscatus	MPEG 5548	-1.065952°	-46.789510°
M. l. lemniscatus	MPEG 5568	-1.192609°	-46.138680°
M. l. lemniscatus	MPEG 8851	-5.369967°	-49.116928°
M. l. lemniscatus	MPEG 8853	-1.065952°	-46.789510°
M. l. lemniscatus	MPEG 8878	-1.024141°	-46.653811°
M. l. lemniscatus	MZUSP Xingu2010MB053	-2.177222°	-52.255000°
M. l. lemniscatus	NHMW 13384:2	-15.598917°	-56.094894°
M. l. lemniscatus	AMNH 57285	-4.592412°	-74.574855°
M. l. lemniscatus	MPEG 8887	-1.065952°	-46.789510°
M. l. lemniscatus	USNM 566289	3.752793°	-59.310080°
M. l. lemniscatus	AMNH 67958	-1.236390°	-69.921890°
M. l. lemniscatus	FMT 1200	-3.060700°	-60.013000°
M. l. lemniscatus	IBSP 47039	-3.768933°	-49.673651°
M. l. lemniscatus	MPEG 10118	-1.816667°	-46.283333°
M. l. lemniscatus	MPEG 12889	-1.816667°	-46.283333°
M. l. lemniscatus	MPEG 13645	-1.520533°	-52.581468°
M. l. lemniscatus	MPEG 2285	-1.192609°	-46.138680°
M. l. lemniscatus	MPEG 8845	-1.522752°	-46.903334°
M. l. lemniscatus	MPEG 8879	-1.024141°	-46.653811°
M. l. lemniscatus	MPEG 8888	-1.065952°	-46.789510°
M. l. lemniscatus	FMT 230	-2.034352°	-60.025875°
M. l. lemniscatus	MPEG 1037	-1.192609°	-46.138680°
M. l. lemniscatus	MPEG 14141	-1.192609°	-46.138680°
M. l. lemniscatus	MZUSP 8042	-3.060700°	-60.013000°
M. l. lemniscatus	MZUSP Xingu2010MC027	-2.184333°	-52.270600°
M. l. lemniscatus	FMT 2425	-3.060700°	-60.013000°

M. l. lemniscatus	FMT 2723	-3.060700°	-60.013000°
M. l. lemniscatus	MPEG 14927	-1.192609°	-46.138680°
M. l. lemniscatus	MPEG 15279	-1.192609°	-46.138680°
M. l. lemniscatus	MPEG 16198	-3.464106°	-44.863080°
M. l. lemniscatus	MPEG 17513	-2.034352°	-60.025875°
M. l. lemniscatus	MPEG 20498	-1.080973°	-46.989449°
M. l. lemniscatus	MPEG 5602	-1.065952°	-46.789510°
M. l. lemniscatus	MZUSP 9097	-3.900000°	-52.666667°
M. l. lemniscatus	FMT 2649	-3.060700°	-60.013000°
M. l. lemniscatus	FMT 325	-2.034352°	-60.025875°
M. l. lemniscatus	IBSP 46798	-3.768933°	-49.673651°
M. l. lemniscatus	MPEG 13004	-1.065952°	-46.789510°
M. l. lemniscatus	MPEG 14513	-4.296611°	-47.556608°
M. l. lemniscatus	MPEG 23720	-1.520533°	-52.581468°
M. l. lemniscatus	MPEG 3906	-1.024141°	-46.653811°
M. l. lemniscatus	MNHN 1996.7849	5.383333°	-52.950000°
M. l. lemniscatus	MPEG 15552	-5.703804°	-48.174719°
M. l. lemniscatus	MPEG 8891	-1.192609°	-46.138680°
M. l. lemniscatus	FMT 1304	-0.405060°	-63.082900°
M. l. lemniscatus	MPEG 18745	-0.942019°	-47.117876°
M. l. lemniscatus	FMT 1914	-3.060700°	-60.013000°
M. l. lemniscatus	FMT 34	-2.034352°	-60.025875°
M. l. lemniscatus	MPEG 3135	-1.192609°	-46.138680°
M. l. lemniscatus	MPEG 3138	-1.192609°	-46.138680°
M. l. lemniscatus	MPEG 5021	-1.065952°	-46.789510°
M. l. lemniscatus	MPEG 5546	-1.065952°	-46.789510°
M. l. lemniscatus	MZUSP 17319	-8.781712°	-63.702289°
M. l. lemniscatus	MPEG 8889	-1.192609°	-46.138680°
M. l. lemniscatus	FMT 674	-3.060700°	-60.013000°
M. l. lemniscatus	MPEG 17606	-5.818016°	-46.144142°
M. l. lemniscatus	FMT 2819	-3.060700°	-60.013000°
M. l. lemniscatus	MPEG 13517	-3.461651°	-44.784000°
M. l. lemniscatus	MPEG 18696	-1.455020°	-48.502368°
M. l. lemniscatus	MPEG 12694	-5.150000°	-44.966667°
M. l. lemniscatus	MPEG 6833	-1.746440°	-47.065910°
M. l. lemniscatus	MPEG 8837	-1.816667°	-46.283333°
M. l. lemniscatus	MNHN 1973.0342	-1.455020°	-48.502368°
M. l. lemniscatus	MPEG 8892	-1.192609°	-46.138680°
M. l. lemniscatus	MPEG 1358	-1.192609°	-46.138680°
M. l. lemniscatus	FMT 2695	-3.060700°	-60.013000°
M. l. lemniscatus	MPEG 18963	-1.805694°	-50.714616°
M. l. lemniscatus	MPEG 10121	-1.192609°	-46.138680°
M. l. lemniscatus	MPEG 1322	-1.192609°	-46.138680°
M. l. lemniscatus	CEPB (S65)	-8.781712°	-63.702289°
M. l. lemniscatus	MPEG 22827	-1.884701°	-48.765215°
M. l. lemniscatus	IBSP 47181	-3.768933°	-49.673651°
M. l. lemniscatus	MNHN 1900.0476	-3.361133°	-64.672629°
M. l. lemniscatus	MPEG 8466	-1.361334°	-48.245425°
M. l. lemniscatus	MPEG 22054	-1.805694°	-50.714616°
M. l. lemniscatus	MPEG 22184	-6.497371°	-49.878436°
M. l. lemniscatus	MPEG 20530	-3.396434°	-43.554185°
M. l. lemniscatus	MPEG 6551	-1.024141°	-46.653811°
M. l. lemniscatus	MPEG 11291	-2.134507°	-47.555753°
M. l. lemniscatus	MPEG 5542	-1.816667°	-46.283333°
M. l. lemniscatus	AMNH 14145	6.383886°	-58.695553°
M. l. lemniscatus	AMNH 23290	-1.676488°	-78.648585°

M. l. lemniscatus	ANSP 11630	-3.328687°	-71.854448°
M. l. lemniscatus	MPEG 11107	-5.150000°	-44.966667°
M. l. lemniscatus	MPEG 14134	-1.192609°	-46.138680°
M. l. lemniscatus	MPEG 15026	-3.461651°	-44.784000°
M. l. lemniscatus	MPEG 8849	-1.024141°	-46.653811°
M. l. lemniscatus	FMNH 37434	-1.790082°	-73.188411°
M. l. lemniscatus	MPEG 10178	-5.466667°	-43.300000°
M. l. lemniscatus	MPEG 1511	-1.192609°	-46.138680°
M. l. lemniscatus	MPEG 8838	-1.816667°	-46.283333°
M. l. lemniscatus	UMMZ 59772	-11.476228°	-67.239346°
M. l. lemniscatus	MPEG 10117	-1.192609°	-46.138680°
M. l. lemniscatus	MPEG 13652	-2.544874°	-45.776422°
M. l. lemniscatus	AMNH 36168	-1.398258°	-61.972192°
M. l. lemniscatus	BMNH 1920.1.20.1381	4.889220°	-52.314743°
M. l. lemniscatus	MPEG 18444	-1.518908°	-48.617001°
M. l. lemniscatus	MNHN 1989.3080	4.868835°	-53.017827°
M. l. lemniscatus	MPEG 16162	-3.461651°	-44.784000°
M. l. lemniscatus	MPEG 2856	-0.959779°	-47.495857°
M. l. lemniscatus	IBSP 53259	-2.034352°	-60.025875°
M. l. lemniscatus	MPEG 11285	-2.134507°	-47.555753°
M. l. lemniscatus	IBSP 24851	3.831487°	-51.835444°
M. l. lemniscatus	IBSP 47298	-3.768933°	-49.673651°
M. l. lemniscatus	MPEG 19303	-1.455020°	-48.502368°
M. l. lemniscatus	USNM 164215	8.200000°	-59.780000°
M. l. lemniscatus	AMNH 15221	-1.676488°	-78.648585°
M. l. lemniscatus	IBSP 46796	-3.768933°	-49.673651°
M. l. lemniscatus	MPEG 20913	-7.143833°	-55.377521°
M. l. lemniscatus	AMNH 14144	6.383886°	-58.695553°
M. l. lemniscatus	IBSP 47297	-3.768933°	-49.673651°
M. l. lemniscatus	IBSP 47771	-3.768933°	-49.673651°
M. l. lemniscatus	IBSP 46251	-9.973999°	-67.807568°
M. l. lemniscatus	MPEG 11570	-1.192609°	-46.138680°
M. l. lemniscatus	MPEG 8852	-1.065952°	-46.789510°
M. l. lemniscatus	AMNH 14150	6.383886°	-58.695553°
M. l. lemniscatus	FMT 2256	-3.566944°	-66.966944°
M. l. lemniscatus	IBSP 47041	-3.768933°	-49.673651°
M. l. lemniscatus	MPEG 24098	-1.518908°	-48.617001°
M. l. lemniscatus	ZSM 648/20	-1.884701°	-48.765215°
M. l. lemniscatus	AMNH 57286	-4.592412°	-74.574855°
M. l. lemniscatus	BMNH 1843.1.10.5	6.804496°	-58.154883°
M. l. lemniscatus	IBSP 46764	-3.768933°	-49.673651°
M. l. lemniscatus	MPEG 23799	-5.370657°	-49.118699°
M. l. lemniscatus	MPEG 3669	-1.065952°	-46.789510°
M. l. lemniscatus	IBSP 47044	-3.768933°	-49.673651°
M. l. lemniscatus	IBSP 47320	-3.768933°	-49.673651°
M. l. lemniscatus	IBSP 48302	-3.768933°	-49.673651°
M. l. lemniscatus	IBSP 48953	-3.768933°	-49.673651°
M. l. lemniscatus	MPEG 4146	-0.930660°	-47.385646°
M. l. lemniscatus	BMNH 1882.1.22.14	7.250000°	-58.716667°
M. l. lemniscatus	IBSP 13777	3.831487°	-51.835444°
M. l. lemniscatus	IBSP 46168	-3.768933°	-49.673651°
M. l. lemniscatus	IBSP 47676	-3.768933°	-49.673651°
M. l. lemniscatus	MPEG 16025	-1.192609°	-46.138680°
M. l. lemniscatus	MPEG 19814	-6.497371°	-49.878436°
M. l. lemniscatus	IBSP 46935	-3.768933°	-49.673651°
M. l. lemniscatus	MPEG 22052	-1.805694°	-50.714616°

M. l. lemniscatus	MPEG 23079	-2.169679°	-56.742140°
M. l. lemniscatus	MPEG 4319	-1.816667°	-46.283333°
M. l. lemniscatus	AMNH 14149	6.383886°	-58.695553°
M. l. lemniscatus	FMT 709	-3.060700°	-60.013000°
M. l. lemniscatus	MPEG 14882	-1.192609°	-46.138680°
M. l. lemniscatus	ZMH 2700	-11.743568°	-67.095233°
M. l. lemniscatus	MPEG 18961	-1.960333°	-48.196543°
M. l. lemniscatus	MPEG 8885	-1.806851°	-46.319225°
M. l. lemniscatus	MPEG 9492	-5.722099°	-50.725419°
M. l. lemniscatus	MPEG 3904	-1.024141°	-46.653811°
M. l. lemniscatus	MPEG 5562	-1.192609°	-46.138680°
M. l. lemniscatus	MPEG 8454	-1.065952°	-46.789510°
M. l. lemniscatus	FMT 583	-2.034352°	-60.025875°
M. l. lemniscatus	MPEG 16199	-3.464106°	-44.863080°
M. l. lemniscatus	MPEG 16313	-1.192609°	-46.138680°
M. l. lemniscatus	MPEG 21559	-1.759944°	-55.862470°
M. l. lemniscatus	MPEG 22053	-1.805694°	-50.714616°
M. l. lemniscatus	MPEG 5020	-1.065952°	-46.789510°
M. l. lemniscatus	MPEG 8877	-1.024141°	-46.653811°
M. l. lemniscatus	USNM 145461	6.804496°	-58.154883°
M. l. lemniscatus	FMT 278	-3.060700°	-60.013000°
M. l. lemniscatus	MPEG 10122	-1.192609°	-46.138680°
M. l. lemniscatus	MPEG 16296	-1.192609°	-46.138680°
M. l. lemniscatus	MPEG 16489	-5.370657°	-49.118699°
M. l. lemniscatus	MPEG 5390	-1.024141°	-46.653811°
M. l. lemniscatus	MPEG 5603	-1.065952°	-46.789510°
M. l. lemniscatus	MPEG 8848	-1.024141°	-46.653811°
M. l. lemniscatus	MCZ 152650	5.224289°	-52.753213°
M. l. lemniscatus	MPEG 10119	-1.816667°	-46.283333°
M. l. lemniscatus	MPEG 20127	-1.805694°	-50.714616°
M. l. lemniscatus	IBSP 51378	-2.034352°	-60.025875°
M. l. lemniscatus	MPEG 4464	-1.192609°	-46.138680°
M. l. lemniscatus	MPEG 10116	-1.024141°	-46.653811°
M. l. lemniscatus	MPEG 13763	-1.816667°	-46.283333°
M. l. lemniscatus	MPEG 15147	-5.150000°	-44.966667°
M. l. lemniscatus	MPEG 16488	-6.003539°	-50.292969°
M. l. lemniscatus	MPEG 6552	-1.024141°	-46.653811°
M. l. lemniscatus	MPEG 3712	-1.192609°	-46.138680°
M. l. lemniscatus	FMT 2237	-3.060700°	-60.013000°
M. l. lemniscatus	FMT 2047	-3.060700°	-60.013000°
M.potyguara	NRM 1685	-8.057581°	-34.888922°
M.potyguara	UFPB 4358	-7.115320°	-34.861051°
M.potyguara	UFPB 4359	-7.115320°	-34.861051°
M.potyguara	UFPB 4355	-7.115320°	-34.861051°
M.potyguara	CAS 49297	-6.127257°	-35.165177°
M.potyguara	UFPB 4361	-7.115320°	-34.861051°
