# Supplementary material for: Reducing Wallacean shortfalls for the coralsnakes of the Micrurus lemniscatus species complex: Present and future distributions under a changing climate
Source: PLoS One. 2018 Nov 14;13(11):e0205164. doi: 10.1371/journal.pone.0205164 (PMC6241113; doi:10.1371/journal.pone.0205164)
Supplement: S2 Table — Number of occurrence records from museum collections and after mapping over the South American grid. (PDF) [file pone.0205164.s004.pdf]

S2 Table. Number of occurrence records. Number of occurrence records from museum collections and after mapping over the South American grid.

Species	Museum records	Grid records
M.l. lemniscatus	264	75
M. l. carvalhoi	289	97
M. diutius	124	33
M. l. helleri	85	49
M. potyguara	6	3
