# Supplementary material for: Reducing Wallacean shortfalls for the coralsnakes of the Micrurus lemniscatus species complex: Present and future distributions under a changing climate
Source: PLoS One. 2018 Nov 14;13(11):e0205164. doi: 10.1371/journal.pone.0205164 (PMC6241113; doi:10.1371/journal.pone.0205164)
Supplement: S3 Table — Details of the climatic simulations (AOGCMs) used in the ecological niche modeling. (PDF) [file pone.0205164.s005.pdf]

S3 Table. Climatic models. Details of the climatic simulations (AOGCMs) used in the ecological niche modeling.

Model ID	Modeling Center	Resolution*	Source	Year
CCSM4	University of Miami – RSMAS. USA	0.9° × 1.25°	CMIP5/PMIP3	2012
CNRM-CM5	Centre National de Recherches Meteorologiques / Centre Europeen de Recherche et Formation Avancees en Calcul Scientifique. France	1.4° x 1.4°	CMIP5/PMIP3	2012
GISS-E2-R	NASA Goddard Institute for Space Studies. USA	2.5° x 2.0°	CMIP5/PMIP3	2012
MIROC-ESM	Atmosphere and Ocean Research Institute (University of Tokyo). National Institute for Environmental Studies. and Japan Agency for Marine-Earth Science and Technology. Japan	2.8° × 2.8°	CMIP5/PMIP3	2012
MRI-CGCM3	Meteorological Research Institute. Japan	1.1° x 1.1°	CMIP5/PMIP3	2012

* longitude × latitude

CMIP5 – Coupled Model Intercomparison Project. Phase 5 (http://cmip-pcmdi.llnl.gov/)

PMIP3 – Paleoclimate Modelling Intercomparison Project. Phase 3 (http://pmip3.lsce.ipsl.fr/)
