# Supplementary material for: Reducing Wallacean shortfalls for the coralsnakes of the Micrurus lemniscatus species complex: Present and future distributions under a changing climate
Source: PLoS One. 2018 Nov 14;13(11):e0205164. doi: 10.1371/journal.pone.0205164 (PMC6241113; doi:10.1371/journal.pone.0205164)
Supplement: S4 Table — Loadings of the bioclimatic variables in the first five axes of varimax rotated factor analysis, based on the CCSM AOGCM. Numbers in bold highlight the highest loading of the selected variable in each factor. (PDF) [file pone.0205164.s006.pdf]

S4 Table. Output for factorial analysis. Loadings of the bioclimatic variables in the first five axes of varimax rotated factor analysis, based on the CCSM AOGCM. Numbers in bold highlight the highest loading of the selected variable in each factor.

Bioclimatic variables	I	II	III	IV	V
**1**	**0.967**	0.088	0.158	0.154	0.096
2	0.024	-0.027	0.288	-0.835	0.290
3	0.330	0.275	0.142	0.178	0.529
4	-0.352	-0.345	-0.087	-0.655	-0.489
5	0.942	0.062	0.207	-0.237	-0.047
6	0.861	0.165	0.080	0.460	0.112
**7**	-0.172	-0.169	0.120	**-0.939**	-0.216
8	0.893	-0.008	0.117	-0.047	0.112
9	0.847	0.159	0.154	0.353	0.052
10	0.977	-0.009	0.142	-0.074	-0.095
11	0.894	0.181	0.162	0.310	0.213
12	0.107	0.900	-0.216	0.073	0.335
**13**	0.151	**0.940**	0.137	0.108	0.240
**14**	-0.267	0.087	**-0.921**	0.133	0.000
15	0.258	0.184	0.787	0.004	0.188
16	0.113	0.931	0.133	0.047	0.282
17	-0.128	0.227	-0.909	0.177	0.146
**18**	-0.149	0.383	-0.132	-0.117	**0.666**
19	0.042	0.590	-0.182	0.188	-0.171

1.Annual Mean Temperature; 2.Mean Diurnal Range (Mean of monthly (max temp - min temp)); 3.Isothermality; 4.Temperature Seasonality (standard deviation *100); 5.Max Temperature of Warmest Month; 6.Min Temperature of Coldest Month; 7.Temperature Annual Range; 8.Mean Temperature of Wettest Quarter; 9.Mean Temperature of Driest Quarter; 10.Mean Temperature of Warmest Quarter; 11.Mean Temperature of Coldest Quarter; 12.Annual Precipitation; 13.Precipitation of Wettest Month; 14.Precipitation of Driest Month; 15.Precipitation Seasonality (Coefficient of Variation); 16.Precipitation of Wettest Quarter; 17.Precipitation of Driest Quarter; 18.Precipitation of Warmest Quarter; 19.Precipitation of Coldest Quarter.
