# Supplementary material for: Reducing Wallacean shortfalls for the coralsnakes of the Micrurus lemniscatus species complex: Present and future distributions under a changing climate
Source: PLoS One. 2018 Nov 14;13(11):e0205164. doi: 10.1371/journal.pone.0205164 (PMC6241113; doi:10.1371/journal.pone.0205164)
Supplement: S5 Table — Ecological niche modeling methods used to estimate species potential distributions. (PDF) [file pone.0205164.s007.pdf]

S5 Table. Ecological niche modeling methods. Ecological niche modeling methods used to estimate species potential distributions.

Method	Species data type
Bioclimatic Envelope (Bioclim)	Presence only
Ecological Niche Factor Analysis (ENFA)	Presence only
Euclidian Distance (EuclidDist)	Presence only
Flexible discriminant analysis (FDA)	Presence and absence
Generalized additive models (GAM)	Presence and absence
Generalized Linear Models (GLM)	Presence and absence
Gower Distance (GowerDist)	Presence only
Mahalanobis Distance (MahalDist)	Presence only
Multivariate adaptive regression splines (MARS)	Presence and absence
Maximum Entropy (Maxent)	Presence/background
Neural Networks (NNET)	Presence and absence
Random Forest (RNDFOR)	Presence and absence
