# Supplementary material for: Reducing Wallacean shortfalls for the coralsnakes of the Micrurus lemniscatus species complex: Present and future distributions under a changing climate
Source: PLoS One. 2018 Nov 14;13(11):e0205164. doi: 10.1371/journal.pone.0205164 (PMC6241113; doi:10.1371/journal.pone.0205164)
Supplement: S6 Table — The mean true skill statistics (TSS) values across five AOGCMs (CCSM, CNRM, GISS, MIROC and MRI) for each ENM method. (PDF) [file pone.0205164.s008.pdf]

S6 Table. Output of model evalution. The mean true skill statistics (TSS) values across five AOGCMs (CCSM, CNRM, GISS, MIROC and MRI) for each ENM method.

**Method/species**	*M. l. lemniscatus*	*M. l. carvalhoi*	*M. diutius*	*M. l. helleri*
BioClim	0.521	0.551	0.535	0.446
ENFA	0.535	0.401	0.507	0.400
EuclidDist	0.474	0.589	0.470	0.333
FDA	0.556	0.621	0.559	0.413
GAM	0.605	0.674	0.626	0.538
GLM	0.579	0.643	0.559	0.506
GowerDist.	0.551	0.599	0.571	0.378
MahalDist	0.561	0.587	0.535	0.425
MARS	0.562	0.643	0.605	0.447
Maxent	0.612	0.640	0.606	0.496
NNET	0.483	0.612	0.555	0.421
RNDFOR	0.583	0.674	0.628	0.582
**Mean**	**0.552**	**0.603**	**0.563**	**0.449**
