# Supplementary material for: Reducing Wallacean shortfalls for the coralsnakes of the Micrurus lemniscatus species complex: Present and future distributions under a changing climate
Source: PLoS One. 2018 Nov 14;13(11):e0205164. doi: 10.1371/journal.pone.0205164 (PMC6241113; doi:10.1371/journal.pone.0205164)

S1 Fig. Habitat suitability maps. Consensus maps of habitat suitability derived from 12 niche modeling methods and 5 climate models. Hot colors indicate high habitat suitability; cool colors, low habitat suitability. Black dots indicate presence records used in the modeling processes; A) *Micrurus l. lemniscatus*, B) *Micrurus l. carvalhoi*, C) *Micrurus diutius*, D) *Micrurus l. helleri*.

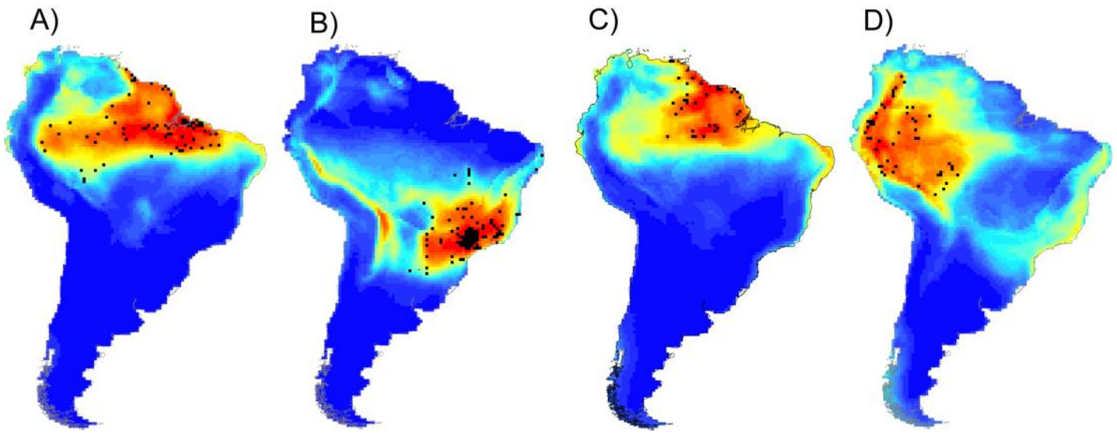

Supplement: S1 Fig — Consensus maps of habitat suitability derived from 12 niche modeling methods and 5 climate models. Hot colors indicate high habitat suitability; cool colors, low habitat suitability. Black dots indicate presence records used in the modeling processes; A) Micrurus l. lemniscatus, B) Micrurus l. carvalhoi, C) Micrurus diutius, D) Micrurus l. helleri. (PDF) [file pone.0205164.s009.pdf]
