# Supplementary material for: Reducing Wallacean shortfalls for the coralsnakes of the Micrurus lemniscatus species complex: Present and future distributions under a changing climate
Source: PLoS One. 2018 Nov 14;13(11):e0205164. doi: 10.1371/journal.pone.0205164 (PMC6241113; doi:10.1371/journal.pone.0205164)

S2 Fig. Habitat suitability map for *M. l. cavalhoi*. Consensus maps of habitat suitability from ENMs for *M. l. cavalhoi* for A) present, and B) future after controlling for spatial autocorrelation in occurrence records. Black dots are the most equidistant records select in the environmental space.

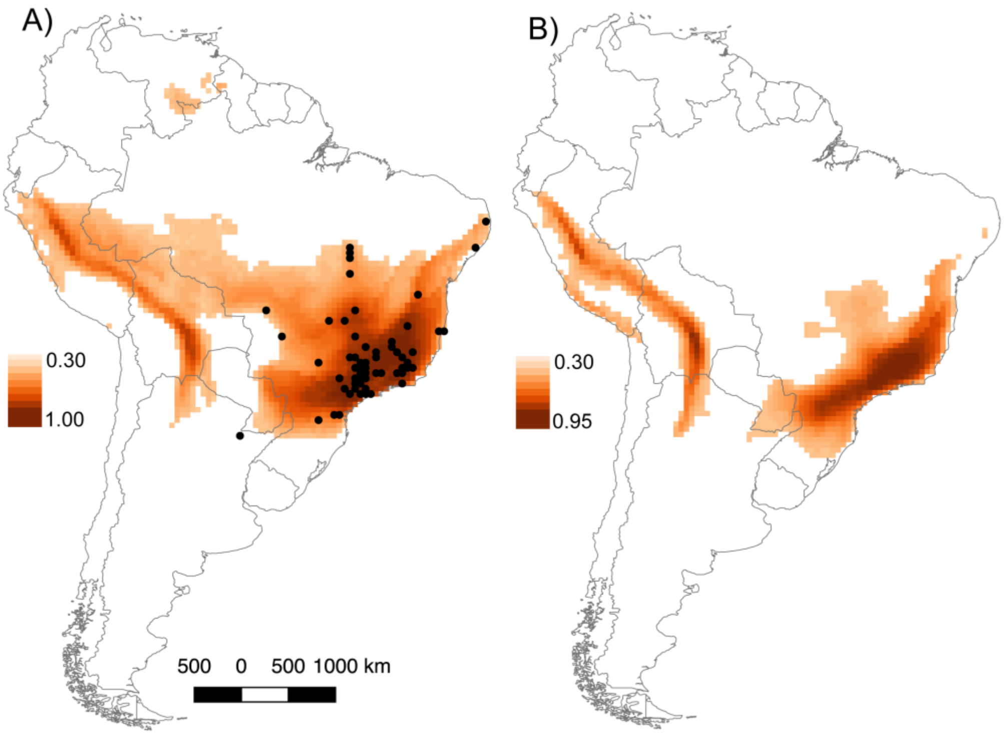

Supplement: S2 Fig — Consensus maps of habitat suitability from ENMs for M. l. cavalhoi for A) present, and B) future after controlling for spatial autocorrelation in occurrence records. Black dots are the most equidistant records select in the environmental space. (PDF) [file pone.0205164.s010.pdf]
