# Supplementary material for: Reducing Wallacean shortfalls for the coralsnakes of the Micrurus lemniscatus species complex: Present and future distributions under a changing climate
Source: PLoS One. 2018 Nov 14;13(11):e0205164. doi: 10.1371/journal.pone.0205164 (PMC6241113; doi:10.1371/journal.pone.0205164)

S3 Fig. **Terrestrial ecoregions.** Delimitation of the terrestrial ecoregions used to assess the level of species representation in protected areas. For each taxon, the selection of these areas was done by overlapping the map of habitat suitability (after applying specific decision thresholds) with the map of South American ecoregions. An ecoregion was selected if at least one cell overlapping it was predicted as present; A) *Micrurus l. lemniscatus*, B) *Micrurus l. carvalhoi*, C) *Micrurus diutius*, D) *Micrurus l. helleri*.

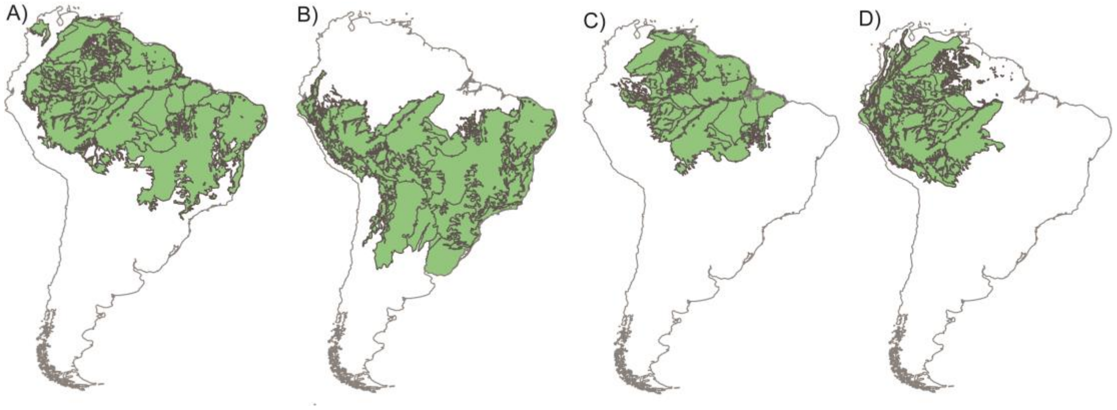

Supplement: S3 Fig — Delimitation of the terrestrial ecoregions used to assess the level of species representation in protected areas. For each taxon, the selection of these areas was done by overlapping the map of habitat suitability (after applying specific decision thresholds) with the map of South American ecoregions. An ecoregion was selected if at least one cell overlapping it was predicted as present; A) Micrurus l. lemniscatus, B) Micrurus l. carvalhoi, C) Micrurus diutius, D) Micrurus l. helleri. (PDF) [file pone.0205164.s011.pdf]
