# Supplementary material for: Reducing Wallacean shortfalls for the coralsnakes of the Micrurus lemniscatus species complex: Present and future distributions under a changing climate
Source: PLoS One. 2018 Nov 14;13(11):e0205164. doi: 10.1371/journal.pone.0205164 (PMC6241113; doi:10.1371/journal.pone.0205164)

S4 Fig. Spatial distribution of the sum of squares. Proportion of the sum of squares (SS) from the ANOVA accounted for by the ecological niche models (ENMs), atmosphere-ocean general circulation models (AOGCMs) and time (the present and the end of the century). Dark red indicates high SS; light red, low SS. A) *Micrurus l. lemniscatus*, B) *Micrurus l. carvalhoi*, C) *Micrurus diutius*, D) *Micrurus l. helleri*.

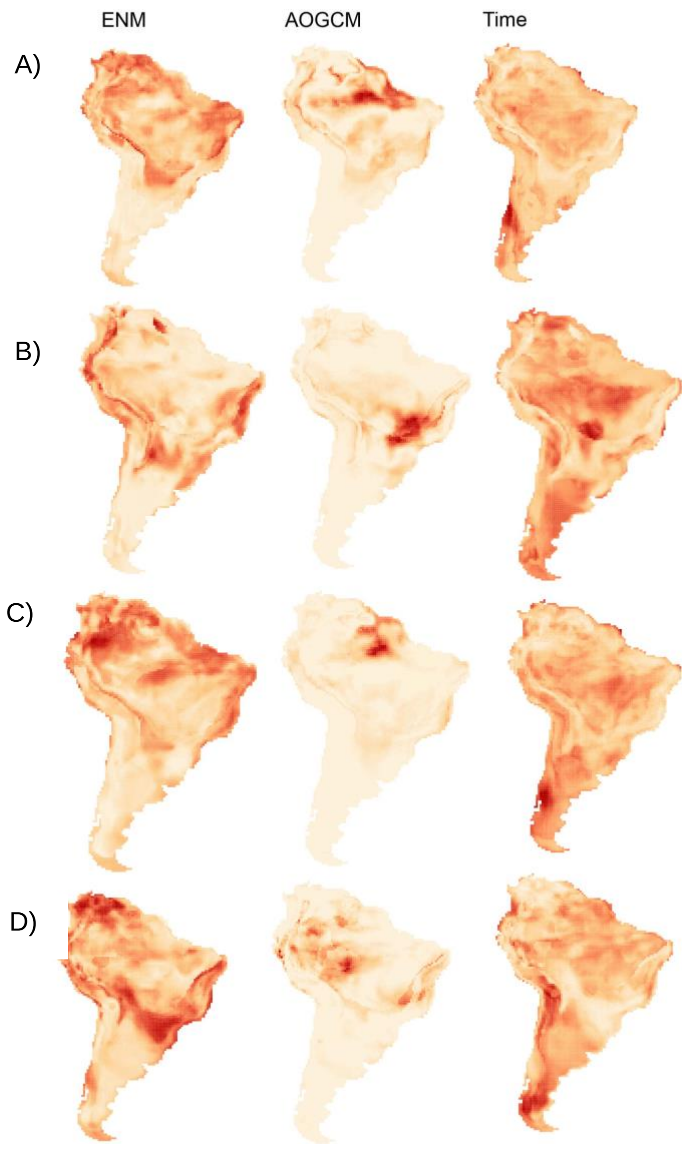

Supplement: S4 Fig — Proportion of the sum of squares (SS) from the ANOVA accounted for by the ecological niche models (ENMs), atmosphere-ocean general circulation models (AOGCMs) and time (the present and the end of the century). Dark red indicates high SS; light red, low SS. A) Micrurus l. lemniscatus, B) Micrurus l. carvalhoi, C) Micrurus diutius, D) Micrurus l. helleri. (PDF) [file pone.0205164.s012.pdf]
